# Biomass-Derived Polysilsesquioxane Nanofilament Reinforced Porous Aerogel for Durable Passive Radiative Cooling across All Day and Weather Conditions

**DOI:** 10.1021/acsnano.5c11008

**Published:** 2025-10-30

**Authors:** Jie Xu, Kangwei Chen, Alessandro Maturilli, Alexandre Laroche, Lingshen Meng, Jörg Knollenberg, Stefan Seeger

**Affiliations:** † Department of Chemistry, 27217 University of Zurich, Winterthurerstrasse 190, CH-8057 Zurich, Switzerland; ‡ Institute for Planetary Research, 54363 German Aerospace Center DLR, Rutherfordstr. 2, 12489 Berlin, Germany

**Keywords:** passive radiative cooling, 1D polysilsesquioxane nanofilaments, aerogel, micro- and nanoporous structure, thermal emission, sunlight reflection, low thermal conductivity

## Abstract

Passive radiative cooling (PRC) is a potentially sustainable strategy by reflecting sunlight (0.3–2.5 μm) and emitting heat through the atmospheric window (8–13 μm) without energy consumption. However, challenges remain due to high sunlight irradiance (1000 W m^–2^) during the day. Our research addresses these challenges by incorporating one-dimensional polysilsesquioxane nanofilaments (1D PSNFs) into micro- and nanoporous biomass-derived aerogels, forming a three-dimensional framework. The designed sustainable aerogel cooler achieves greater than 97% sunlight reflection and thermal emission, resulting in a cooling power of 138.6 W m^–2^ over 720 h, reducing ambient temperatures by 9 °C. In addition, the aerogel cooler demonstrates high thermal stability, low thermal conductivity (29.0 mW m^–1^ K^–1^), superhydrophobicity (water contact angle ∼175°), low density (44.43 kg/m^3^), and a large surface area (137.84 m^2^/g). These features enable effective radiative cooling across various weather conditions, while also maintaining environmental sustainability.

## Graphical abstract

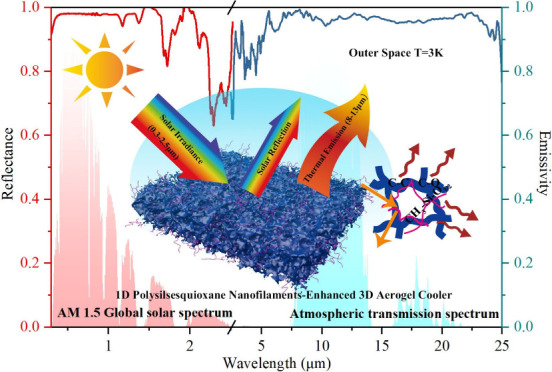

Global warming and energy consumption mutually exacerbate each other, causing irreversible environmental harm. Currently, the Earth’s surface is experiencing exceptionally high summer temperatures.^1,2^ Cooling is particularly important for providing thermal comfort to humans. Nonetheless, overreliance on conventional cooling systems such as air conditioning results in enormous energy consumption, aggravating the greenhouse effect.^3,4^

Passive radiative cooling (PRC) has gained attention as a potentially sustainable alternative with a minimal carbon footprint. PRC involves spontaneous cooling by reflecting sunlight (0.3 to 2.5 μm) and emitting heat directly to cold outer space (*T* = 3 K)^5^ through the atmospheric transparent spectral window in the mid-infrared wavelength range of 8 to 13 μm with zero energy consumption.^6–8^ Despite numerous outstanding research efforts in recent years, there are still many challenges left for PRC materials, considering the sunlight irradiance of 1000 W m^–2^ in the daytime; even minimal solar energy absorption could negate the effects of radiative cooling.^8,9^ Accounting for all heat exchange processes, the net cooling power of a radiative cooler was considered and can be determined as follows:^10–12^

$$P_{cool}\left(T\right)=P_{rad}\left(T\right)-P_{atm}\left(T_{atm}\right)-P_{sun}\left(T\right)-P_{cond+conv}\left(T\right)$$

where *P*_*rad*_ is the output power of the emitter, *P*_*atm*_ is the absorbed radiation power from the ambient, *P*_*sun*_ is the incident solar power absorbed, and *P*_*cond+conv*_ is the loss of cooling power due to convection and conduction. To enhance the net cooling power, one should maximize *P*_*rad*_ while minimizing *P*_*sun*_, *P*_*atm*_, and *P*_*cond+conv*_. Often it is focused on increasing *P*_*rad*_, as it is the only pathway for the emission of radiation.

Since the first multilayer photonic daytime radiative cooler was designed by Fan’s group,^13^ numerous emerging photonic structure designs of radiators have appeared.^10,14^ However, the high cost or fragility of most photonic crystal devices limits their practical applications.^8^ Over the past several years, various polymer-based PRC materials, which offer benefits such as superior storage, ease of processing, affordable transportation, and exceptional cooling performance, have garnered significant attention, such as polymer coatings,^6^ polymer films,^12^ white paints,^15^ artificial woods,^1^ plastics,^16^ and polymer aerogels.^17^ Among them, polymer aerogels have seen increased use as PRC materials, given that their ordered symmetrical micropores and randomized nanopores exhibit high solar reflectance due to total internal reflection minimizing *P*_*sun*_ and *P*_*atm*_.^18–21^ In addition, specific chemical bonds in the polymers (C–C, C–F, and C–O) emit thermal energy through the atmospheric transmission window at mid-infrared wavelengths.^16,22^ Their ultralow density, low thermal conductivity, hydrophobicity, high surface area, and high porosity contribute to a reduced *P*_*cond+conv*_, enabling effective performance in real-world applications.^23^ In contrast, polymer-based films, coatings, or paints are often vulnerable to UV-induced degradation, contamination, and mechanical failure, whereas polymer aerogels retain both high radiative efficiency and low thermal conductivity, making them a more durable and stable PRC solution.^24–26^ Leroy et al.^27^ developed a polyethylene aerogel (PEA) with outstanding properties including a solar-weighted reflectance of 92.2% (6 mm thickness), infrared transmittance of 79.9% in the 8–13 μm band (6 mm), low thermal conductivity (28 mW·m^–1^·K^–1^), and a daytime cooling power of 96 W/m^2^ and achieved up to 13 °C subambient cooling around solar noon. Peng et al.^28^ designed a scalable low-carbon ambient-dried foam-like aerogel offering scalable, low-carbon fabrication and excellent durability under harsh conditions (pH 1–13, high temperature), with good solar reflectance (93%) and infrared emissivity (94%) achieving 4.8 °C cooling.

However, most radiative cooling aerogels are nonrecyclable and are nonrenewable, which necessitates extensive use of limited resources and could potentially be detrimental to the environment.^16,29^ Recently, more and more researchers have focused on biomass-derived greener materials.^30,31^ A cellulose acetate-based bilayer aerogel achieves high solar reflectivity (95.7%), strong emittance (up to 98.7%), and notable cooling performance (12.25 °C outdoors), while maintaining low thermal conductivity and structural elasticity.^32^ To this end, alternative biomass-derived polymers such as poly­(lactic acid) (PLA), which is derived from corn and is industrially scalable, could be explored as eco-friendly replacements. In addition, the cooling performance, mechanical strength, hydrophobicity, and aging resistance of current systems still present opportunities for further optimization through structural and compositional tuning.^33,34^

Moreover, specific chemical bonds that vibrate at 8–13 μm, such as C–OH, −CF_3_, and Si–O–Si, can contribute to improved selective thermal emissivity.^35^ Recently, microparticles of various types and sizes, such as SiO_2_,^36^ CaCO_3_,^37^ Al_2_O_3_,^38^ BaSO_4_,^39^ and TiO_2_,^40^ have been applied through techniques such as painting, self-assembly, spray coating, and embedding in polymers. Integrating nanofilaments into polymers is a promising approach for achieving radiative cooling, due to their large surface area and fibrous frameworks, which serve as excellent supports, allowing for easy modification of the optical, thermal, and other properties of materials.^22,41^

In this study, we developed a biomass-derived 3D aerogel interspersed with 1D PSNFs, featuring micro- and nanoporous structures for effective radiative cooling both day and night with significantly enhanced structure and performance, thanks to the uniform distribution and entanglement of 1D PSNFs within the 3D PLA framework. Consequently, this hierarchical micro- and nanostructure within the aerogel brings many advantages, including higher thermal stability, higher melting point, and water-repellent performance with low thermal conductivity and density, compared with conventional PLA aerogels. We achieved a high sunlight reflection of ≈97%, thermal emission of ≈97%, and stable cooling power of 138.6 W m^–2^ for 720 h continuously under sunlight, resulting in a temperature reduction of approximately 9 °C in the outdoor test. Therefore, we expect to be able to enhance the cooling efficiency during hot summers. Unlike other polymer PRC materials, the 1D PSNF-interspersed micro- and nanoporous PLA 3D aerogel cooler (PSNF/MNPLA) consistently exhibits a high WCA of ∼175°. It maintains its optical and cooling efficiency, regardless of reductions in thickness, exposure to chemical corrosion, or outdoor weathering under natural sunlight, showing great potential as an eco-friendly all-day passive radiative cooler in diverse weather scenarios.

## Results and Discussion

Recently, the hierarchical porous polymer coatings, films, and foams have shown great potential in radiative cooling.^6,7,42,43^ However, there is still much room for improvement in their reflectivity, emissivity, and mechanical properties to achieve a leap in cooling performance. Achieving this goal with only polylactic acid (PLA) is rather difficult, so we introduced one-dimensional polysilsesquioxane nanofilaments (1D PSNFs) in hierarchal nano–microstructured PLA to obtain a reinforced nanocomposite aerogel. First, we synthesized the 1D PSNFs through liquid phase decomposition using toluene as the reaction medium and methyltrichlorosilane (MTCS) as the precursor; the details of the method can be found in the Methods, with the corresponding synthesis mechanism also shown (Figure S1).

To fabricate the porous structure, we uniformly dispersed the prepared 1D PSNFs in a mixture of dioxane and water. Through solvent-assisted thermally induced phase separation,^6,45,46^ micro- and nanoscale pores were formed within the PLA and 1D PSNFs composite. Due to the different solubility and miscibility characteristics of PLA in dioxane and water, where PLA is insoluble in water but readily dissolves in dioxane, it forms a continuous phase in the dioxane. Because dioxane is fully miscible with water and has a lower freezing point, phase separation occurs during freezing. Larger ice crystals form first, followed by nanoscale ice crystals that develop on the PLA framework. 1D PSNFs, insoluble in both dioxane and water, exist as dispersed nanofilaments that cannot be incorporated into the crystal lattice. As ice forms, the freezing front physically expels the 1D PSNFs into the remaining liquid channels, concentrating them at PLA-rich/ice-rich interfaces.

This behavior is consistent with the freeze-casting mechanism described by Shao et al.^47^ Solidification induces phase separation in which suspended particles are typically rejected by the advancing solid–liquid interface if the interfacial free energy balance satisfies

$$\Delta \gamma_{0}=\Delta \gamma_{ps}-\Delta \gamma_{pl}+\Delta \gamma_{sl}>0$$

where γ_ps_, γ_pl_, and γ_sl_ are particle–solid, particle–liquid, and solid–liquid interfacial energies. Capillary forces and thermodynamic phase separation further drive their localization along pore boundaries—energetically favorable sites for separating water from the superhydrophobic PSNFs. Upon freeze-drying, the ice is removed and the 1D PSNFs are effectively “locked” at the pore walls. The differential sublimation of dioxane and water effectively preserves the intricate micro- and nanostructured porous network, as shown in Figure 1a.^45^ Such stereoscopic interspersed 1D PSNFs in porous PLA skeletons can be beneficial for enhancing the thermal properties and other functionalities of the PLA aerogel for passive radiative cooling applications when compared with the original PLA aerogel. We can see from Figure 1b and c that a superhydrophobic (CA ∼ 175°) cooler with a very high average reflectivity (97%) and average emissivity (97%) was obtained. Compared with the other PRC materials in the references (Figure 1d), the cooler from this work maintains its competitiveness because it can simultaneously possess high reflectivity and emissivity as well as cooling temperature, which results in a higher daytime cooling power at ambient temperature (138.6 W m^–2^ at 40 °C).

**Figure 1:**
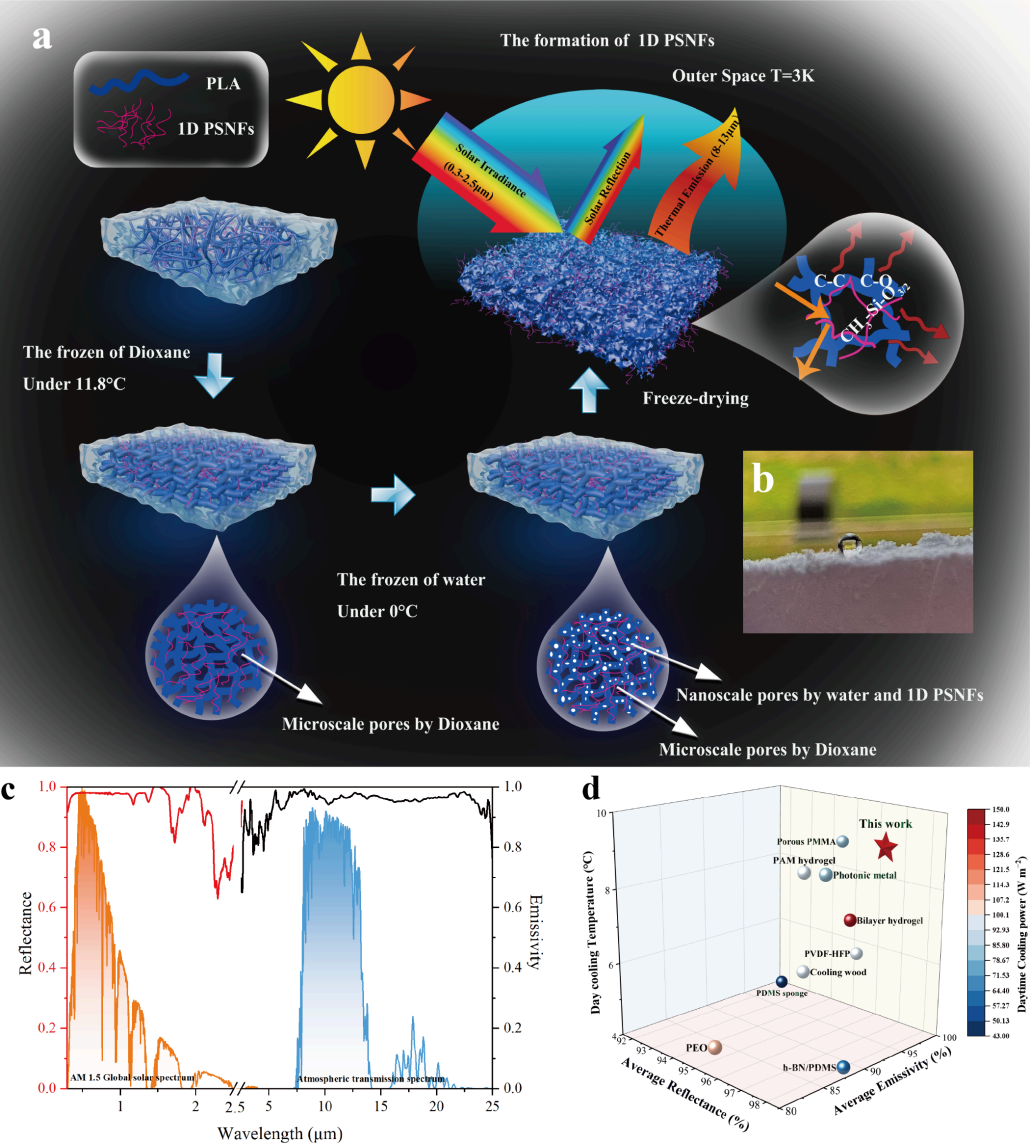
a) Fabrication process and schematic of the formation of a 1D PSNF-interspersed micro- and nanoporous PLA 3D aerogel cooler (PSNF/MNPLA). b) Image of a water droplet on the composite aerogel. c) Reflectance and emissivity spectra of PSNF/MNPLA in the 0.25–25 μm wavelength range against the normalized ASTM G-173-03 AM1.5 global spectrum (as standardized in ISO 9845-1, 1992)^32,44^ and the atmospheric transparency spectrum. d) Comparison of this work with current PRC materials in the literature in terms of average solar reflectance, mid-infrared emissivity, daytime cooling temperature, and daytime cooling power.

To clarify the difference in the structure and morphology of the pristine PLA aerogel and the 1D PSNF-interspersed hierarchical nano–microporous 3D PLA aerogel (PSNF/MNPLA), we used a high-resolution scanning electron microscope (SEM). We obtained images at a different magnification and measured the corresponding pore size distribution using Nano Measurer software (Figure 2a and b). Upon examination, we can only find micropores in the PLA aerogel. They exhibited a broad pore size distribution ranging from 30 to 110 μm and an average microsized pore with a diameter of 66.45 ± 18.11 μm (Figure 2b). However, no distinct nanosized pores are discernible in the PLA aerogel, even under higher magnification in SEM images. In contrast, the MNPLA aerogel exhibits significantly smaller microsized pores (6.85 ± 2.25 μm), along with the formation of new nanosized pores embedded within the PLA skeleton. With the incorporation of 1D PSNFs, both micro- and nanoscale pore sizes in the PSNF/MNPLA aerogel are further reduced—from 6.85 ± 2.25 μm to 5.42 ± 1.68 μm for micropores and from 509.25 ± 204.98 nm to 341.37 ± 185.18 nm for nanopores.

**Figure 2:**
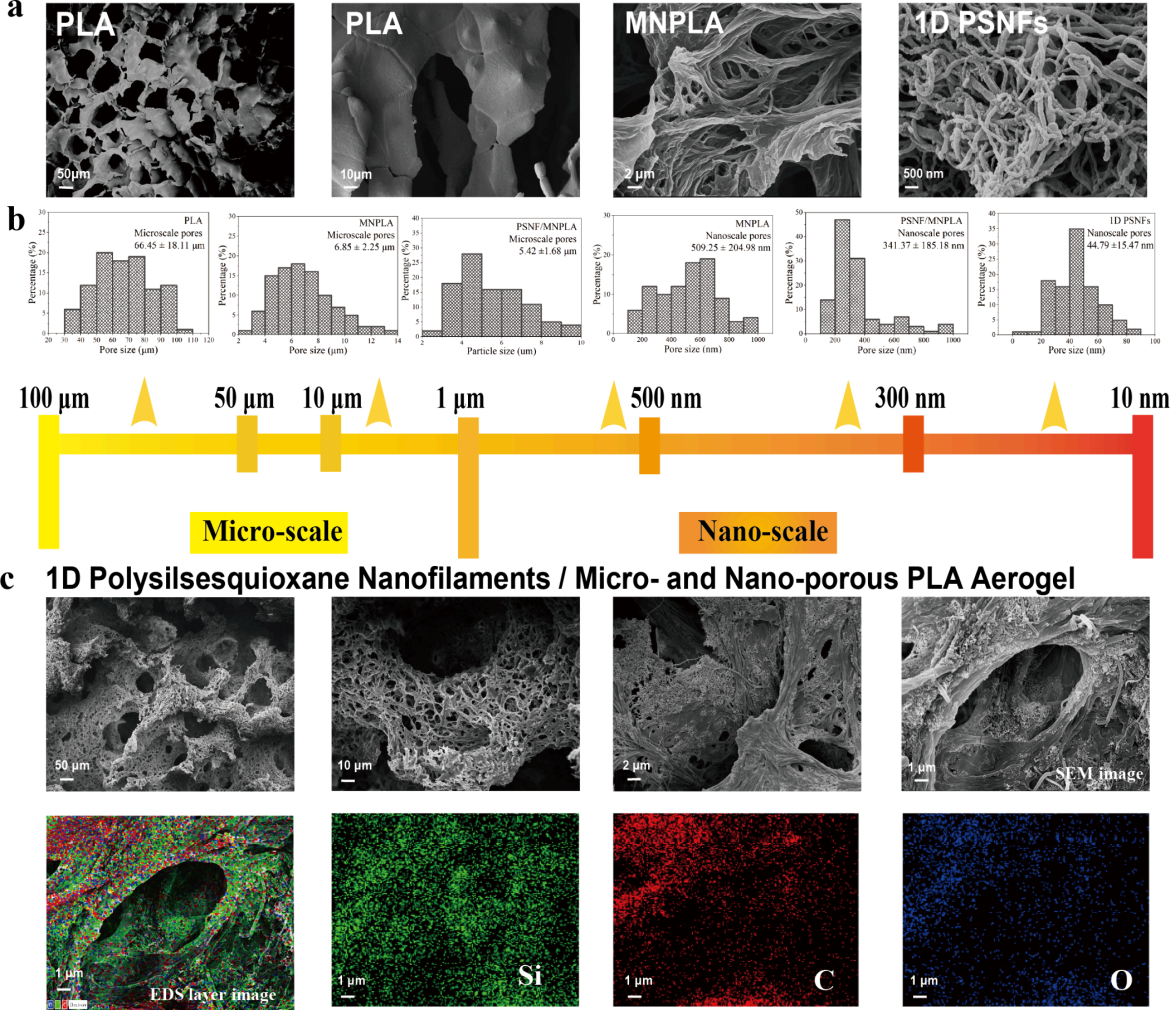
a) SEM images of PLA aerogel, MNPLA, and 1D PSNFs (isolated). b) Corresponding micro- and nanopore size distribution of PLA, MNPLA, PSNF/MNPLA aerogels, and 1D PNFs measured by Nano Measurer. c) SEM image and its EDX mapping of the layer image and Si, C, and O elements of the PSNF/MNPLA aerogel.

And the SEM images (Figure 2c) distinctly showcase the 1D PSNFs intricately interlacing and encircling nano- and microsized pores, resulting in a reduction of pore size. This is due to the inherent structure and slender diameter of the 1D PSNFs (55.62 ± 15.65 nm), creating many smaller nanopores (44.79 ± 15.47 nm) (Figure S2).

Therefore, embedding 1D PSNFs into the porous skeleton forms new nanopores with some polymer fibers or pores, while retaining existing pores. Energy-dispersive X-ray (EDX) was used to obtain images proving that the introduced 1D PSNFs are distributed and wrapped around the micro- and nanosized pores of the PLA aerogel skeleton (Figure 2c). From the EDX layer images, the distribution of Si, exclusive to the 1D PSNFs, is distinctly visible. An interesting phenomenon is that the Si elements are primarily localized at the edges and within the interior of the pores; this is because the superhydrophobicity of 1D PSNFs favors the polymer-rich side, but they cannot fully merge on either the polymer-rich side or the polymer-poor side; thus, we can observe many 1D PSNFs gathering at the edges of the pores. In addition, the hierarchical porous structured skeleton can be a very good holder, which allows the interspersion of the 1D PSNFs inside.

Daytime radiative cooling fundamentally hinges on spectrum selection; the main task is to maximize solar reflection and thermal emission power simultaneously.^17^ Figure 1c demonstrated the spectral reflectance and emissivity of the PSNF/MNPLA aerogel cooler with 3 mm effective thickness according to the AM 1.5 global solar spectrum and the atmospheric transparency window. With approximately 95.8% porosity the hierarchical micro- and nanostructures present a high average reflectance (97%) in the solar spectral (0.3–2.5 μm) region and high average emissivity (0.97) in the atmospheric transparent window (8–13 μm). The higher solar reflection of the micro- and nanoporous PLA (MNPLA) aerogel (88%) than the PLA aerogel (73%) is attributed to the sunlight scattering of micro- and nanopores with broadly distributed sizes.^48^ Increasing the quantity of 1D PSNFs leads to a reduction in nanopore size, decreasing from 509.25 ± 204.98 nm to 341.37 ± 185.18 nm. This results in a marked enhancement in solar reflection, moving from 88% (for MNPLA) to 97% (for PSNF/MNPLA) by introducing 1D PSNFs, as shown in Figure 3a and c. A transparent window with high emissivity in the atmosphere is essential for transferring heat to the cold universe. Thanks to the chemical bonds C–O and C–C from PLA, all of the aerogels showed a relatively high emissivity at the atmospheric transparent window (Figure 3b).

**Figure 3:**
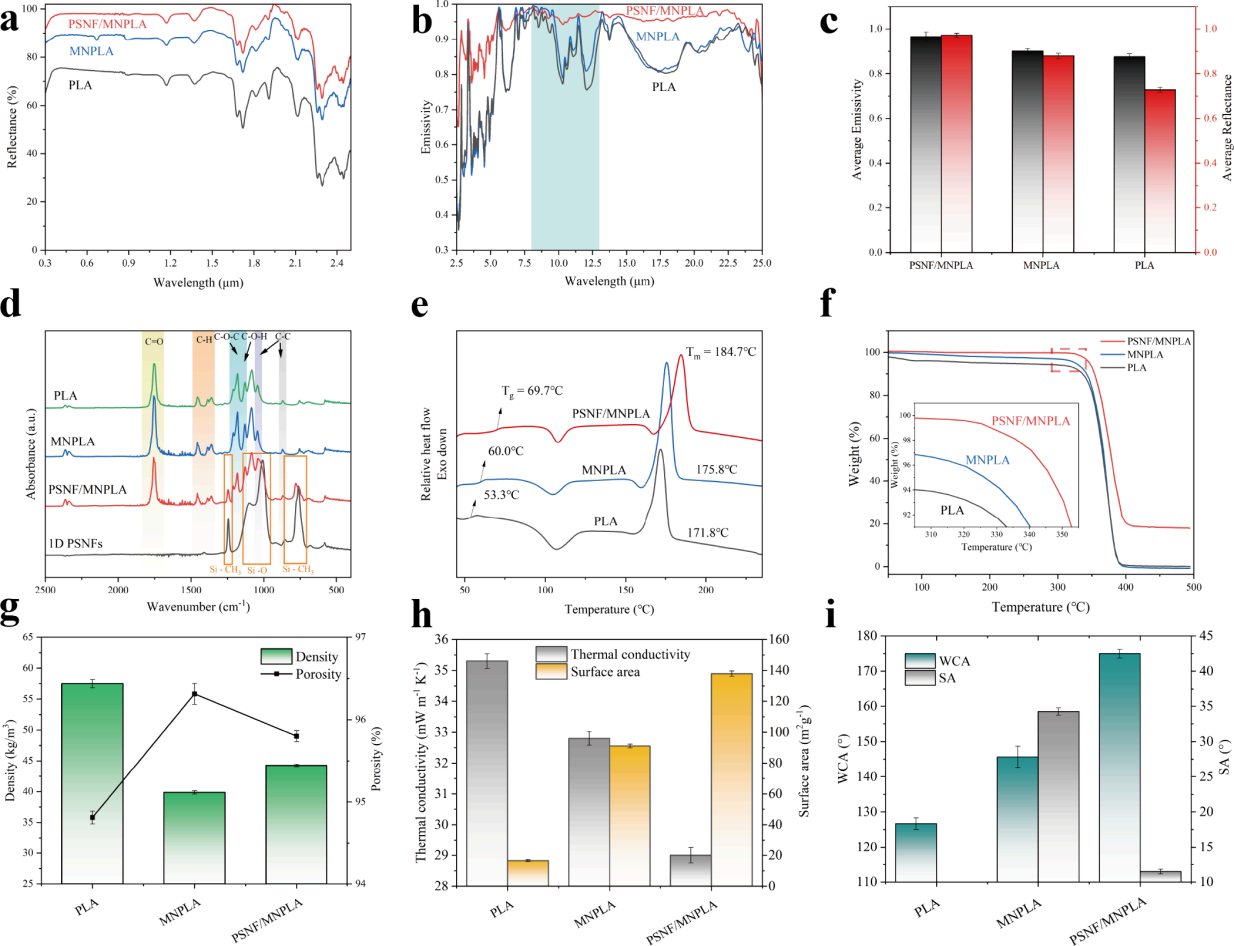
a) Reflectance, b) emissivity, c) average reflectance and emissivity (8–13 μm), d) absorbance spectra, e) DSC curves, f) TGA curves, g) density and porosity, h) thermal conductivity and surface area, and i) water contact angle of the aerogels (PLA, MNPLA, and PSNF/MNPLA aerogels).

In addition, finite-difference time-domain (FDTD) simulations were performed to further analyze the influence of the pore size on the optical performance (Figure S3 and Figure S4). In the UV–vis–NIR range (Figures S3a and S3b), decreasing the diameter of micropores led to enhanced solar reflectance, reaching a peak at around 5 μm pore size. Similarly, nanopores in the range of 50–500 nm exhibited a significant impact on light scattering: the reflectance generally increased with a reduction in pore size. In the mid-infrared region, high emissivity was maintained across most nanopore sizes (Figure S3c), with only minor spectral deviations. However, the micropore size played a more pronounced role: micropores (5–10 μm) exhibited higher emissivity in the 8–13 μm atmospheric transparency window (Figure S3d), which is essential for passive radiative cooling. The simulation results are consistent with our experimental observations, supporting the idea that the hierarchical porous structure of the PSNF/MNPLA aerogel—comprising both nanoscale pores (341.37 ± 185.18 nm) and microscale pores (5.42 ± 1.68 μm)—is responsible for the enhanced broadband reflectance across the entire solar and infrared spectrum.

The absorbance spectra of samples were determined using ATR-FTIR (attenuated total reflectance Fourier-transform infrared spectroscopy) and are displayed in Figure 3d, showing a carbonyl vibration C=O at 1748 cm^–1^, stretching of C–O at 1181 and 1130 cm^–1^, and stretching of C–C at 1046 and 872 cm^–1^ belonging to PLA.^49^ The absorption peaks of 1D PSNFs at 1100 cm^–1^ and 1009 cm^–1^ correspond to the stretching vibration of the Si–O–Si bond, 780 cm^–1^ belongs to −CH_3_ rocking and Si–C stretching in Si–CH_3_, and 1244 cm^–1^ is attributed to CH_3_ bending in Si–CH_3_.^50,51^ All of these absorption peaks fall between 770 and 1250 cm^–1^ (8–13 μm) within the atmospheric transparency window. By incorporation of 1D PSNFs, the intensity of absorption peaks associated with Si–O–Si and Si–CH_3_ increases, leading to a higher emissivity (0.97) from 1D PSNF enhanced MNPLA compared to 0.90 from pure MNPLA (Figure 3b and c). Therefore, the combination of PLA and 1D PSNFs can significantly enhance the emissivity of the biomass-derived aerogel cooler in the atmospheric transparent window and its excellent reflectivity in the solar spectrum. However, as the quantity of 1D PSNFs increases from 0% to 30%, the reflectance values initially rise sharply, reaching their peak at 20% 1D PSNFs (97.17%), after which they decline. Similarly, their emissivity initially increases and reaches its maximum value at 20% 1D PSNFs (96.57%), remaining stable at 30% 1D PSNFs (96.41%) (Figure S5a to e). These findings suggest that interspersing 20% 1D PSNFs is the optimal amount for achieving effective all-day passive radiative cooling.

The enhancement of the thermal properties of the aerogel coolers was also determined by differential scanning calorimetry (DSC) and thermogravimetric analysis (TGA). Figure 3e shows the DSC traces of quenched samples obtained at a heating rate of 10 °C/min, while Table S1 summarizes the detailed thermal performance parameters (*T*_g_, *T*_m_, *T*_d5_, *T*_d10_, *T*_max_, and *R*_500_) obtained. As can be observed, neat PLA aerogel presents a heat enthalpy change located at 53.3 °C related to the glass transition (*T*_g_); an exothermic peak was observed due to cold crystallization at 106.8 °C,^52^ followed by a melting peak (*T*_m_) at 171.8 °C. It is worth mentioning that both *T*_g_ and *T*_m_ of samples increased thanks to their micro- and nanopores in MNPLA (60.0 and 175.8 °C). The interspersing of 1D PSNFs can also increase *T*_g_ and *T*_m_, reaching 69.7 and 184.7 °C, respectively. In the TGA curves (Figure 3f), incorporating 1D PSNFs produces a nanocomposite with a more stable mass-loss profile than the original PLA aerogel below 300 °C. As the 1D PSNFs content increases from 0 to 30 wt %, *T*_d5_ rises significantly (from 212.1 to 350.1 °C), while *T*_max_ shows only slight fluctuations (Table S1). The residue at 500 °C also increases, reflecting the intrinsic thermal robustness of 1D PSNFs, which maintain structural integrity with negligible weight loss up to 500 °C. This enhanced thermal stability reduces the risk of thermal deformation or collapse under prolonged solar exposure, thereby preserving the aerogel’s optical and mechanical performance during outdoor radiative cooling.

Due to the formation of a microporous structured skeleton in the PLA aerogel, it features inherently low density (57.50 ± 0.66 kg/m^3^) and porosity (94.8%). Creating the nanoscale pores in the aerogel effectively led to a decrease in the density of MNPLA to 37.86 ± 0.26 kg/m^3^ and an increase in the porosity to 96.3%. The density and porosity changed slightly as the added 1D PSNFs increased. They wrapped around the pores, modifying the size of both nano- and microsized pores and resulting in a relatively lower porosity (95.8%). Concurrently, owing to the substantial surface area of 1D PSNFs (295.9 ± 3.16 m^2^/g), their inclusion played a pivotal role in amplifying the surface area of the PLA aerogel from an initial 16.49 ± 0.67 m^2^/g, reaching 137.84 ± 1.72 m^2^/g when 20% 1D PSNFs were incorporated (Figure 3g). As an effective cooling material, strong thermal insulation is essential to minimize heat gain through conduction. The porous architecture of the PLA aerogel inherently contributes to a reduced thermal conductivity of 35.3 mW·m^–1^·K^–1^. With the incorporation of 1D PSNFs and the formation of a hierarchical micro/nanoporous network, the thermal conductivity is further reduced to 29.0 mW·m^–1^·K^–1^ (Figure 3h). This enhancement is attributed to the increased phonon scattering, elongated heat transfer paths, and interfacial thermal resistance introduced by the complex multiscale structure.^53^ Furthermore, polymers generally have surfaces that are easily wetted and contaminated; PLA surfaces tend to show high adhesion to water droplets.^54^

Even with the aid of micro- and nanostructures, the MNPLA aerogel can only achieve a water contact angle (WCA) of 145.6° ± 3.06° and a sliding angle (SA) of 34.2° ± 0.52°. As the amount of 1D PSNFs was increased, the WCA of the aerogels reached 175.0° ± 1.22°. There was also a significant decrease in SA, reaching 11.5° ± 0.33° (Figure 3i), which, according to the increased roughness of the surface, indicated improved water-repellent properties.

To experimentally confirm that the 1D PSNFs aerogel cooler preserves its excellent radiative cooling performance, we made a setup (Figure 4a) and performed continuous outdoor measurements on pure PLA, MNPLA, and PSNF/MNPLA aerogel samples on a sunny day during the summer in Zurich, Switzerland (Figure 4b). Three samples were placed in the same experimental devices under the same air temperature and solar irradiance. All temperatures were recorded (Figure 4d and g), and the cooling temperatures of the samples for both nighttime and daytime were plotted to demonstrate further the cooling performance (Figure 4e and h).

**Figure 4:**
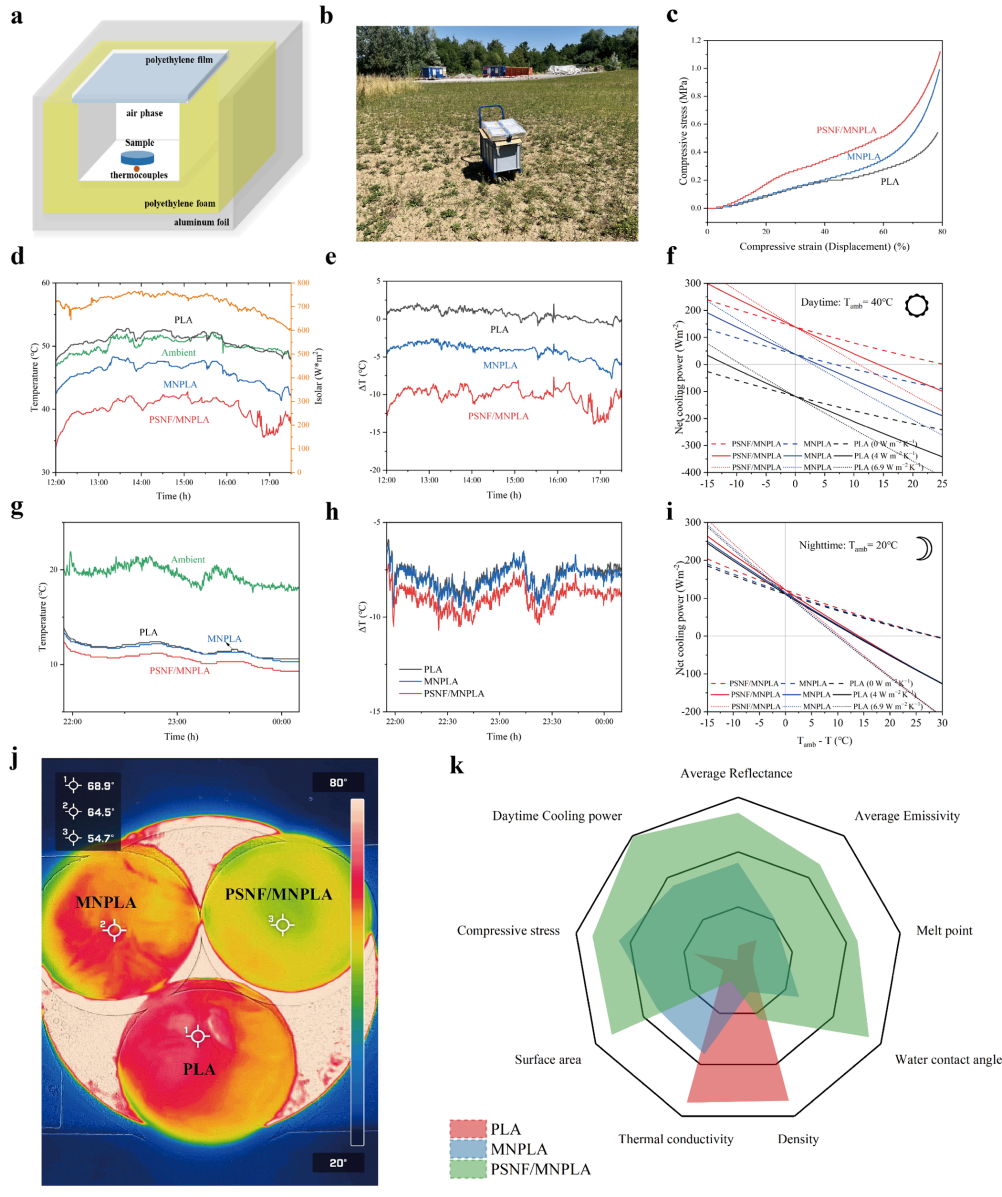
a) Schematic of the setup for testing performance under sunlight. b) Photos from the outdoor test. c) Compressive stress–strain curves of aerogels undergoing 80% strain. d, g) temperature and e, h) temperature difference of PLA, MNPLA, and PSNF/MNPLA aerogels in the daytime (conducted on July 7, 2023, under clear skies and low wind conditions; during the test period, average ambient temperatures were 44.8 °C, with average relative humidity of 35%) and nighttime (conducted on July 9–10, 2023, under clear skies and low wind conditions; during the test period, ambient temperatures were around 24.3 °C, with average relative humidity of 65%) in Zurich, Switzerland. f, i) Their calculated cooling power for daytime (40 °C) and nighttime (20 °C) with *h*_c_ = 0, 4, and 6.9 W m^–2^ K^–1^. n) The infrared thermal image of aerogel heating at *T* = 100 °C for 10 min. o) Radar plots comparing the performance of PLA, MNPLA, and PSNF/MNPLA samples.

In the daytime (from 12:00 to 17:30), under sunlight, the ambient temperature was as high as ≈48.2 °C, and the temperature of the PLA aerogel reached an average temperature of around 48.7 °C, slightly above the ambient temperature. Due to its hierarchical micro- and nanoporous structure effectively blocking radiative heat transfer, the MNPLA aerogel maintained an average temperature of 44.0 °C, slightly below the ambient temperature. Notably, the temperature of the PSNF/MNPLA composite aerogel maintained a lower temperature of 39.2 °C, consistently below the ambient temperature, showing a subambient temperature reduction of approximately 9 °C (as seen in Figure 4e). The significant temperature reduction during the daytime in PSNF/MNPLA is attributed to its high sunlight reflectivity, strong emissivity in the atmospheric transparency window, and low thermal conductivity due to the interlaced 1D PSNFs inside the aerogel skeleton, which reduce the size of both micro- and nanopores while increasing the number of nanosized pores. The other aerogels experienced heat accumulation, because of their inability to reflect or dissipate heat. Furthermore, all samples showed a significant temperature drop at night (≈19.5 °C, 11.6 °C, 11.5 °C, and 10.5 °C for ambient, PLA, MNPLA, and PSNF/MNPLA, respectively). In comparison (Figure 4h), the differences in their corresponding cooling temperature between samples at nighttime are lower than in the daytime (−7.8 °C, −8.0 °C, and −9.1 °C for PLA, MNPLA, and PSNF/MNPLA, respectively). During the nighttime, as the measured solar irradiance values were near zero, the radiative cooling performances for each radiative cooler were higher than those in the daytime.^55^

Additionally, the theoretical net cooling power of the aerogel radiative coolers for different nonradiative heat coefficient (*h*_c_) can be determined using equations S3–S9. The *P*_*cooling*_ value when *T*_*amb*_ – *T*_*coole*__r_ = 0 is described as the net cooling power at the ambient air temperature unaffected by nonradiative heat. Even under the solar irradiance of about 800 W m^–2^ at noon, the corresponding net cooling power of PSNF/MNPLA reached as high as 138.6 W m^–2^ at *T*_*amb*_ = 40 °C = 313.15 K (where *T*_*amb*_ – *T*_*cooler*_ = 0) (Figure 4f). However, the *P*_*cooling*_ values of MNPLA and PLA aerogels are notably low, registering at 37.7 W m^–2^ and −117.7 W m^–2^, respectively. Meanwhile, the Δ*T* value when *P*_*cooling*_ = 0 represents the highest attainable cooling temperature, decreasing with the increasing *h*_c_ values.^56^ In the daytime (40 °C), the maximum temperature drops of PSNF/MNPLA calculated from the *h*_c_ = 0, 4, and 6.9 W m^–2^ K^–1^ are 25.0 °C, 10.9 °C, and 8.0 °C, respectively. In the nighttime, in the absence of solar irradiance, the net cooling power of PSNF/MNPLA, MNPLA, and PLA reached 121.7 W m^–2^, 114.0 W m^–2^, and 110.4 W m^–2^ at an ambient temperature of 20 °C (Figure 4i). The corresponding maximum temperature drops of PSNF/MNPLA are shown to be relatively higher compared with those in the daytime (28.0 °C, 14.1 °C, and 10.4 °C when *h*_c_ are 0, 4 W m^–2^ K^–1^, and 6.9 W m^–2^ K^–1^, respectively). When we decrease the ambient temperature to 300 K, the lower cooling value will be 108.0 W m^–2^ for PSNF/MNPLA (Figure S7). This indicates that the ambient temperature directly affects the cooling power. Adding 1D PSNFs results in radiative coolers with better cooling performance on warm summer days. The infrared images and movie were obtained by placing the coolers on a hot plate set at 100 °C to continue measuring for 10 min. We collected the images at 0, 2, and 10 min (Figure 4j and Figure S9), and they showed that the PSNF/MNPLA aerogel cooler consistently maintained a temperature difference of 14 °C lower than the PLA aerogel. These changes can also be observed in Movie S1, which started recording for 90 s after placing the samples. The radar plot (Figure 4 h) compares the three aerogel coolers. The novel aerogel cooler, which integrates 1D PSNFs into hierarchical porous structures, shows significant performance enhancements, a high average reflectivity, and a high average emissivity. These result in a higher daytime cooling power at ambient temperature (138.6 W m^–2^) than the original PLA (−117.7 W m^–2^). In the meantime, it is also a water-resistant bulk material (CA ∼ 175°) with a high melting point (184.7 °C), low density (44.43 kg/m^3^), lower thermal conductivity (29.0 mW m^–1^ K^–1^), high surface area (137.84 m^2^/g), and a high axial compressive strength because of embedded polysilsesquioxane nanofilaments.

To evaluate whether the hierarchical porous structure and embedded 1D PSNFs reduce the sensitivity of cooling performance to thickness, the reflectance and emissivity spectra of the PLA, MNPLA, and PSNF/MNPLA aerogels with two different thicknesses (3 and 7 mm) were measured (Figure 5a and b). It was shown that the higher thickness would cause higher reflectance of PLA and MNPLA aerogels (Figure 5a). This indicates that the enhanced thickness effectively extends the path of sunlight into the space below, leading to increased light scattering at the polymer–air interfaces, resulting in less heat gain.^21,57^

**Figure 5:**
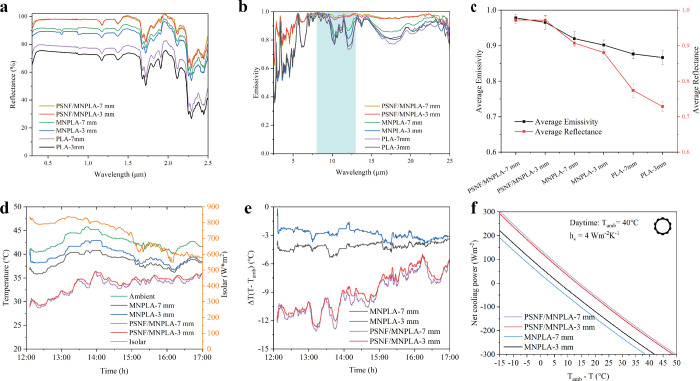
a) Reflectance spectra, b) emissivity spectra, and c) the average reflectance and emissivity of PLA, MNPLA, and PSNF/MNPLA aerogel coolers with 3 mm and 7 mm thickness within the 0.25–25 μm wavelength range. d) Measured temperature and e) temperature difference between coolers and ambient air in the daytime (conducted on July 18, 2023, under clear skies and large wind conditions; during the test period, the average ambient temperature was 41.9 °C, with average relative humidity of 41%) in Zurich, Switzerland. f) Calculated cooling power as a function of cooling temperature for daytime with *h*_c_ = 4 W m^–2^ K^–1^.

However, with the help of 1D PSNFs, even at a reduced thickness, the PSNF/MNPLA aerogel cooler can still reflect the entirety of the solar irradiance, displaying nearly identical average reflectivity values of 0.97 across two different thicknesses (Figure 5c). However, the varying thicknesses of all the aerogel coolers slightly influence the IR emissivity (Figure 5b). In fact, the emissivity was calculated based on Kirchoff’s law: ε­(λ) = 100% – τ­(λ) – ρ­(λ).^58^ Since the transmittance of aerogel samples is very low, the IR emittance is mainly attributed to the vibration of the C–O–C, C–O–H, C–C, Si–CH_3_, and Si–O bonds in the aerogel cooler, indicating the change of thickness does not affect the absorption of all the bonds.^57^ We also measured the absorbance spectra of the inside and outside of the PSNF/MNPLA aerogel cooler at 3 and 7 mm, as shown in Figure S10a. The peak positions and intensities of these samples were similar. Their internal and external appearances also maintained remarkable similarity when the samples were of different thicknesses according to their SEM images (Figure S10b). It indicated the possibility of achieving a very high reflectance and emissivity with a lower thickness. The unique hierarchical porous structure resulting from the stereoscopic interspersed 1D PSNFs introduced a finer scale of micropores (6.85 ± 2.25 μm) and nanopores (341.37 ± 185.18 nm). These pores, combined with the absorption properties of their chemical bonds, contributed to this capability.

The MNPLA and PSNF/MNPLA with thicknesses of 3 and 7 mm were selected for subsequent outdoor tests to measure their cooling performance. However, the weather changed from clear to cloudy, accompanied by intermittent strong winds. Therefore, the temperature and solar irradiance curve fluctuated (Figure 5d). Despite the nonideal weather, the MNPLA and PSNF/MNPLA aerogel samples showed a similar pattern in cooling temperature changes. Their average cooling temperatures were 2.9 and 8.9 °C, respectively, when the average ambient temperature was 42.6 °C. The cooling temperature is lower than the data on 8/7/2023 because of the temperature and wind of the day. Due to the higher reflectivity and emissivity of the thicker sample (7 mm), they have a relatively higher cooling temperature (4.2 and 9.3 °C, respectively). With an increase in thickness, the cooling temperature of the MNPLA aerogel showed a more significant increase (1.3 °C), while the PSNF/MNPLA composite aerogel only increased by 0.3 °C. As we know, the thicker the radiative coolers, the higher the corresponding cost. Thus, a PSNF/MNPLA aerogel cooler of 3 mm thickness was chosen instead of 7 mm, as it offers high reflectivity (97%) and emissivity (97%), resulting in a cooling temperature similar to that of the thicker alternative.

PRC materials under outdoor conditions have stability issues.

Long-term ultraviolet radiation from sunlight can cause a decrease in the reflectivity. Corrosion by acidic or alkaline solutions can damage the structure of the material, making the PRC ineffective.^9^ Additionally, to assess the chemical corrosion resistance of the PSNF/MNPLA aerogel cooler, it was submerged in solutions with pH levels of 1 and 13 for 7 days. To test the outdoor stability of the aerogel coolers, we exposed them to the summer sky in Zurich for 7 days. These tests were conducted to compare the performance of the cooler to that of its initial state.

Both solar reflectance and infrared emittance of the PSNF/MNPLA aerogel coolers have very slight differences for the aerogel coolers submerged in pH = 1 solution and from the outdoor exposure (Figure 6a and b), and their corresponding SEM images are shown in Figure S11. However, the samples immersed in the pH 13 solution displayed a decreased average emissivity of 0.95 in the atmospheric transparency window, whereas the initial samples exhibited an emissivity of 0.97 (Figure 6c). Thanks to the protection of 1D PSNFs, the ATR-FTIR spectra further demonstrate that the surface of the cooler before and after treatment exhibits pronounced absorption peaks with comparable intensities (Figure 6d). The coolers treated differently in outdoor tests showed similar temperatures, as expected (Figure S12a). To more clearly observe the outdoor temperature changes, Figure 6e illustrates the temperature difference between the treated samples and their initial state. The sample exposed to sunlight displays a consistent and similar outcome to that of the initial state. Immersion in a solution with a pH of 1 also results in a temperature that fluctuates closely with the original. In contrast, the temperature difference is slightly increased when immersed in a solution with a pH of 13. We also calculated the corresponding cooling power in the daytime and nighttime, and comparable results were shown among all the samples (Figure S12 b and c). It is also worth noting that the WCA and SA after weathering and solution (acid and base) treatment reflect exceptional durability and potential applicability. By exposure to sunlight, the composite aerogel cooler maintained high stability (Figure 6f). The WCA ranged from 174.99° ± 1.22° to 174.23° ± 0.48°, and the SA varied slightly from 11.50° ± 0.33° to 11.82° ± 0.18° under neutral conditions. Immersion in different pH solutions, however, produced contrasting effects. In the acidic solution (pH = 1), both WCA and SA showed only minor changes, indicating that the 1D PSNFs on the aerogel surface maintained high chemical stability because the Si–O–Si network is less prone to hydrolysis in acidic media. In contrast, in the alkaline solution (pH = 13), more noticeable variations were observed: the WCA decreased to 169.33° ± 0.87°, while the SA increased significantly to 20.18° ± 0.99°. These changes are attributed to partial alkaline hydrolysis of the siloxane bonds (Si–O–Si) within the 1D PSNFs, forming hydrophilic silanol groups (Si–OH) and slightly reducing the intrinsic hydrophobicity of the 1D PSNFs. In addition, localized hydrolysis can cause subtle rounding of nanoscale asperities, weakening the Cassie–Baxter wetting state and increasing droplet adhesion, thereby elevating the SA.

**Figure 6:**
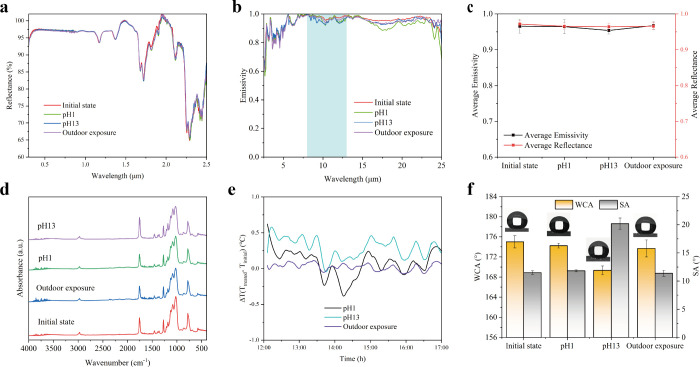
a) Reflectance spectra, b) emissivity spectra, c) average reflectance and emissivity, and d) absorbance spectra of the PSNF/MNPLA aerogel cooler in its initial state after 7 days immersed in pH = 1 and pH = 13 solutions and exposed to the summer sky in Zurich, Switzerland. e) Temperature difference between coolers and ambient air in the daytime (18 July 2023) in Zurich, Switzerland, and h) water contact angle and sliding angle value of PSNF/MNPLA composite aerogel coolers after immersion and outdoor exposure.

In this regard, the PSNF/MNPLA aerogel cooler is stable in its optical and cooling performance throughout chemical corrosion and outdoor exposure tests. This implies that the cooler showcases outstanding resilience against solvents and weather elements, indicating its substantial potential as a highly durable cooling material.

## Conclusion

To summarize, we prepared high-performance 1D PSNFs-interspersed 3D hierarchical micro- and nanoporous PLA aerogels for all-day passive radiative cooling. Thanks to the stereoscopic incorporation and selective deposition of 1D PSNFs within a nano/microstructured PLA skeleton, both microsized pores (5.42 ± 1.68 μm) and nanosized pores (341.37 ± 185.18 nm) were reduced, achieving a high sunlight reflection of ≈97% and thermal emission of ≈97%, which results in a high cooling power (138.6 W m^–2^) under sunlight. Simultaneously, a very low thermal conductivity (29.0 mW m^–1^ K^–1^) leads to a temperature drop of ≈9 °C during the daytime and nighttime in the outdoor test. Additionally, the material exhibits water-repellent properties with a water contact angle (WCA) of 175°, alongside high thermal stability (with a melting point of 184.7 °C), low density (44.43 kg/m^3^), and a specific surface area (137.84 m^2^/g).

Unlike other polymer passive daytime radiative cooling materials, the cooler in this work maintains a high WCA and consistent optical and cooling performance even when the thickness is reduced or when subjected to chemical corrosion and outdoor exposure tests. It indicates the significant potential of the 1D PSNFs-interspersed aerogel cooler as a promising high-performance, multifunctional, environment-friendly passive radiative cooler for real-world applications, capable of operating in various weather conditions.

## Methods

### Materials

Polylactic acid pellets (Ingeo Biopolymer 4032D) with L/D ratios from 24:1 to 30:1, methyltrichlorosilane (MTCS) (99%), and Dioxane (99.8%) were purchased from Sigma-Aldrich. Toluene (99%) was from Tommen-Furler AG. Milli-Q water was generated using a Simplicity water purification system (Merck Millipore), achieving a resistivity of 18.2 MΩ·cm. One-dimensional polysilsesquioxane nanofilaments (1D PSNFs) were synthesized via a liquid-phase decomposition process using methyltrichlorosilane (MTCS) as the precursor. Toluene served as the organic solvent and was transferred to a reaction vessel, where its humidity was adjusted using a combination of dry and humidified nitrogen. The final water content was determined by using a Coulometric Karl Fischer titrator (DL32, Mettler Toledo). After reaching the desired moisture level, the solution was equilibrated for 1 h. MTCS was then added to the system via a microsyringe (Hamilton) through a septum. The reaction was stirred at 150 rpm using a magnetic stirrer (IKA RCT standard) and allowed to proceed overnight. Upon completion, the resulting 1D PSNFs were isolated by vacuum filtration, followed by sequential washing with dry toluene, ethanol, and deionized water. The purified nanofilaments were then dried in an oven at 50 °C overnight.

### Fabrication of 1D PSNF Micro/Nanoporous PLA Aerogel Cooler

Specified amounts of 1D polysilsesquioxane nanofilaments (1D PSNFs) were ultrasonically dispersed in 1,4-dioxane at 30 °C for 1 h. Subsequently, poly­(lactic acid) (PLA) pellets were added to the dispersion under continuous sonication and stirring to obtain a homogeneous suspension. Deionized water, equivalent to 5 vol % of dioxane, was then slowly introduced into the mixture, followed by an additional hour of ultrasonication to ensure complete dissolution. The resulting suspension was rapidly frozen at −16 °C overnight to promote phase separation and the formation of micro- and nanostructured pores.

The frozen samples were then subjected to freeze-drying (ALPHA 1-2 LDplus, Martin Christ, Germany) at −55 °C and 0.2 Pa for 48 h. Afterward, the aerogels were further dried in an oven at 55 °C overnight prior to characterization. Aerogels containing varying 1D PSNF loadings (0, 10, 20, and 30 wt %) were prepared to evaluate the effect of nanofilament incorporation. For comparison, a pristine PLA aerogel was also fabricated without the addition of 1D PSNFs or deionized water.

## Supplementary Material





## Notes

Characterization; theoretical thermal calculation of the emitter; Figures S1 and S2: 1D PSNF synthesis mechanism; diameter distribution of 1D PSNFs and EDX of PSNF/MNPLA; Figures S3 and S4: FDTD simulation of porous aerogels with different pore diameters; Figure S5: Characterization of the aerogels with interspersing 0, 10%, 20%, and 30% 1D PSNFs; Figures S6 and S7: Tensile stress–strain curves and the cooling power comparison; Figure S8: Comparison with other coolers; Figure S9: Infrared images of coolers; Figure S10: Absorbance and SEM images at different thicknesses; Figures S11 and S12: Morphology, temperature, and cooling power after corrosion and exposure; Figure S13: Passive daytime radiative cooling performance of the aerogel coolers in different locations and weather; Table S1: Thermal parameters of aerogel samples; Table S2: Comparison with other aerogel coolers (PDF)
Movie S1: Thermal infrared video of PLA, MNPLA, and PSNF/MNPLA coolers (MP4)
