# Supplementary material for: Biomass-Derived Polysilsesquioxane Nanofilament Reinforced Porous Aerogel for Durable Passive Radiative Cooling across All Day and Weather Conditions
Source: ACS Nano. 2025 Oct 30;19(44):38395–407. doi: 10.1021/acsnano.5c11008 (PMC12613843; doi:10.1021/acsnano.5c11008)
Supplement: Supplementary file 1 [file nn5c11008_si_001.pdf]

Supporting Information

Biomass-Derived Polysilsesquioxane Nanofilament Reinforced Porous Aerogel for Durable Passive Radiative Cooling Across All Day and Weather Conditions

*Jie Xu[1], Kangwei Chen[1], Alessandro Maturilli[2], Alexandre Laroche[1], Lingshen Meng[1], Jörg Knollenberg[2], and Stefan Seeger[1]**

[1]Department of Chemistry, University of Zurich, Winterthurerstrasse 190, CH-8057 Zurich, Switzerland.

[2]Institute for Planetary Research, German Aerospace Center DLR, Rutherfordstr. 2, 12489 Berlin, Germany

* Corresponding Author: sseeger@chem.uzh.ch

Characterization

Morphological and chemical characterization: To characterize the surface structures of the samples, a high-resolution Scanning Electron Microscope (SEM) combined with energy-dispersive X-ray (EDX) (Gemini SEM 450, Zeiss, Germany) was utilized at an acceleration voltage of 10 kV under the Inlens detector, and an SEM-EDX was used for the elemental analysis. Before the analysis, each sample was sputtered with 10 nm of Platinum using a CCU-010 HV coating unit (Safematic, Switzerland).

Fourier-transform Infrared Spectroscopy (FTIR) (Bruker Optic Gmbh, Germany) equipped with an Attenuated Total Reflectance (ATR) single reflection crystal was used to collect the spectra in the range of 400-4000 cm^{-1} (64 scans), and the background was recorded against air, to measure the chemical characterization of the samples.

The reported density of PLA aerogels was determined from the average density of three samples, and the uncertainty accounts for the accuracy of the measured mass and volume and variation between samples.

The porosity (P) of the samples was determined by immersing them in ethanol using the following equation: [1]

$$P = \frac{V_1}{V_1 + V_2} \qquad (S1)$$

$$V_1 = \frac{m_0 - m}{\rho_l} \qquad (S2)$$

Where V_l represents the pore volume of the material, V_2 is the volume of solid material skeleton, ρ_l denotes the density of ethanol, m_0 signifies the mass of the aerogels after saturation of ethanol, and m indicates the mass of foam samples. The porosity data were obtained by three independent measurements.

Thermal conductivity measurements were performed using a setup comprising a Keithley 2450 source and a custom DLR hot disk probe. The evaluation of the thermal conductivity was done by the transient plane source inversion method according to standard ISO 22007-2. The hot disk probe was placed between two pieces of PLA foam, and a small weight was employed to enhance the thermal contact between the probe and the sample. The measurement uncertainty is estimated to be around 6% (k=2), which is slightly inferior to typical due to the imperfect nature of the sample and the associated higher repeatability error. Furthermore, a high correlation coefficient (> 0.99998) was observed.

Optical measurements: The solar reflectance in the 0.3-2.500 μm range was measured by ultraviolet-visible spectroscopy (UV-vis, UV3600, Shimadzu) equipped with a $BaSO_4$ integrating sphere (ISR-3100). The hemispherical reflectance in the 2.5–25 μm spectral range was measured in vacuum (0.7 mbar) by Fourier transform infrared spectrometer (FTIR, Bruker Vertex 80V)) with gold integrating sphere. A nitrogen-cooled MCT detector (HgCdTe) and a KBr beamsplitter were used, as source a Globar lamp was used. Reference is a gold standard, provided from the same material as the integrating sphere (Spectralon). 1000 consecutive scans from reference and each sample were acquired to improve the SNR. The emissivity is calculated by using the equation $\varepsilon(\lambda) = 100\% - \tau(\lambda) - \rho(\lambda)$, in which the ε, τ, and ρ represent emissivity, reflectance, and transmittance at the wavelength λ, respectively[2].

The Drop Shape Analyzer (DSA100, Kruss, Germany) was utilized to measure the water contact angle (WCA) and sliding angle (SA). Each sample underwent at least three measurements at different positions at ambient temperature (23.0 °C ± 0.2 °C) using Milli-Q water (10 μL). The Laplace-Young fitting method was used for all measurements.

Thermal analysis and mechanical measurement: The melting point of the resins was conducted using Differential Scanning Calorimetry (DSC, DSC 1, Mettler-Toledo (CH)). About 10 mg of the sample was placed in the crucible with nitrogen purge gas at 50 mL/min. Samples were cycled between 40 and 250 °C at a heating/cooling rate of 10 °C/min. Thermogravimetric analysis (TGA) was used to monitor the thermal degradation of the samples was conducted by a simultaneous thermal analyzer (STA 449 F3, Netsch, German); all the samples (5–10 mg) were heated from 50 to 500 °C with a heating rate of 20 °C/min under a high purity nitrogen flow of 50 mL/min.

The infrared thermal image and video were observed using an infrared thermal imaging camera (FLIR One Pro). The coolers were placed on a hot plate set at 100°C for 10 minutes to capture the infrared imagery and videos.

Compression tests were conducted at room temperature using a universal testing instrument (Instron) with a load of 100 N and a compression rate of 5 mm/min undergoing 80% strain. The aerogel samples were cut into cylinders in advance for measuring (h=10 mm and d= 7 mm), underwent at least five specimens to calculate the average compression test value.

To elucidate the optical mechanisms underlying the hierarchical porous structure of the PSNF/MNPLA aerogel, Finite-Difference Time-Domain (FDTD) simulations were performed using Ansys Lumerical FDTD Solutions. Three-dimensional (3D) models were constructed for numerical analysis. A plane wave source was introduced across the spectral range of 0.3 to

25 μm. Periodic boundary conditions were applied along the x- and y-axes, while perfectly matched layers (PMLs) were used along the z-axis to eliminate boundary reflections. The mesh size was set to 0.5 μm in bulk regions and refined to 25 nm in porous domains to ensure computational accuracy. Reflectance was recorded using frequency-domain field monitors. In the mid-infrared region (2.5–25 μm), assuming the aerogel is optically thick and non-transmissive, emissivity was calculated based on reflectance. To explore the influence of pore architecture, random nanoporous models (50–500 nm) and periodic microporous models (1–20 μm) were established, each with a fixed effective thickness of ~10 μm and uniform pore density. A constant porosity of 90% was maintained across all models. As shown in the updated Figure S3 and Figure S4, the simulations reveal enhanced broadband light scattering resulting from the hierarchical pore design, corroborating the experimental observations of high solar reflectance and strong thermal emissivity.

Outdoor radiative cooling performance measurement: We made a device to measure the cooling performance of the radiative coolers on the empty lawn of the University of Zurich. For the setup, we positioned the sample in polystyrene foam boxes ($44 \times 38 \times 4.5$ cm^3) to limit heat loss. The boxes were wrapped with aluminum film to reflect solar power and thermal radiation from the surroundings. All these design elements ensured that the heat transfer process occurred solely through the top opening of the box. Furthermore, an infrared transparent polyethylene film was utilized to seal the airspace above the setup. This was done to provide a shield against convection and conduction with the surrounding environment. A 4-channel K Digital Thermometer (Sefram 9814) was used to detect the real-time temperature of samples and the ambient temperature (one data point for every 10 s). Moreover, solar irradiance and relative humidity were concurrently recorded around the device using a data-logging optical meter (ILT 2400) and a hygrothermometer (switchbot).

Theoretical thermal calculation of the emitter

As mentioned above, the daytime cooling power of a radiative cooler is calculated by the following equation [3]:

$$P_{cool}(T) = P_{rad}(T) - P_{atm}(T_{atm}) - P_{sun}(T) - P_{cond+conv}(T) \qquad (S3)$$

Where P_{rad} (T)

$$P_{rad}(T) = A2\pi \int_0^{\frac{\pi}{2}} \sin\theta \cos\theta d\theta \int_{2.5}^{25} I_{BB}(T,\lambda)\varepsilon(\lambda,\theta) d\lambda \qquad (S4)$$

Here

$$I_{BB}(T,\lambda) = \frac{2hc^2}{\lambda^5} \frac{1}{e^{\frac{hc}{\lambda kT}} - 1} \tag{S5}$$

where h is the Planck constant (6.62607015 × 10^{-34} m^2 kg / s), *c* is the speed of light in vacuum (299792458 m/s), k is the Boltzmann constant (1.380649×10^{-23} J · K^{-1}), and A is the area of the emitter (0.44 m^2).

$$P_{atm}(T_{amb}) = 2\pi \int_0^{\frac{\pi}{2}} d\theta \sin\theta \cos\theta \int_0^{\infty} d\lambda I_{BB}(T_{amb},\lambda)\varepsilon(\lambda,\theta)\varepsilon_{atm}(\lambda,\theta) \tag{S6}$$

The angle-dependent emissivity of the atmosphere is

$$\varepsilon_{atm}(\lambda,\theta) = 1 - t(\lambda)^{\frac{1}{\cos\theta}} \tag{S7}$$

Where t (λ) is the atmospheric transmittance in the zenith direction, which can be

obtained by MODTRAN of Mid-Latitude Summer Atmosphere Model (MODTRAN (spectral.com))

$$P_{sun}(T) = \int_0^{\infty} d\lambda I_{solar}(\lambda)\varepsilon(\lambda,0) \tag{S8}$$

$$P_{cond+conv}(T) = h_c(T_{amb} - T) \tag{S9}$$

Here $h_c = h_{cond} + h_{conv}$ is the collective nonradiative heat coefficient of the emitter.

The ambient temperature of 300 K and nonradiative heat coefficient values of 0, 4 W m^{-2} K^{-1}, and 6.9 W m^{-2} K^{-1} are taken as examples in several reports, where h_c of 0 indicates an ideal thermal insulation case, h_c of 4 W m^{-2} K^{-1} and 6.9 W m^{-2} K^{-1} indicate different parasitic heat loss cases [1-4] .

The cooling temperature was used ($\Delta T = T_{cooler} - T_{ambient}$), which was calculated by extracting the cooling temperature under the condition of $P_{cooling}(T) = 0$.

The average solar reflectivity ($\bar{R}$) in solar spectral range and average thermal emittance ($\bar{\varepsilon}_{IR}$) in the atmospheric transparency window was calculated using:

$$\bar{R} = \frac{\int_{0.3\mu m}^{2.5\mu m} I_{AM1.5}(\lambda)R(\lambda)d\lambda}{\int_{0.3\mu m}^{2.5\mu m} I_{AM1.5}(\lambda)d\lambda} \tag{S10}$$

$$\bar{\varepsilon}_{IR} = \frac{\int_{8\,\mu m}^{13\mu m} I_{BB}(\lambda,T)\varepsilon(\lambda)d\lambda}{\int_{8\,\mu m}^{13\mu m} I_{BB}(\lambda,T)d\lambda} \tag{S11}$$

Stability test: Chemical stability tests were immersed the cooler into solutions of HCl (pH = 1), and NaOH (pH = 13) for one day. The aging tests were also conducted by exposure samples under the sunlight of the summer sky in Zurich for 7 days, and then the wetting properties and the optical and cooling performance were evaluated.

Methyltrichlorsilan (MTCS)

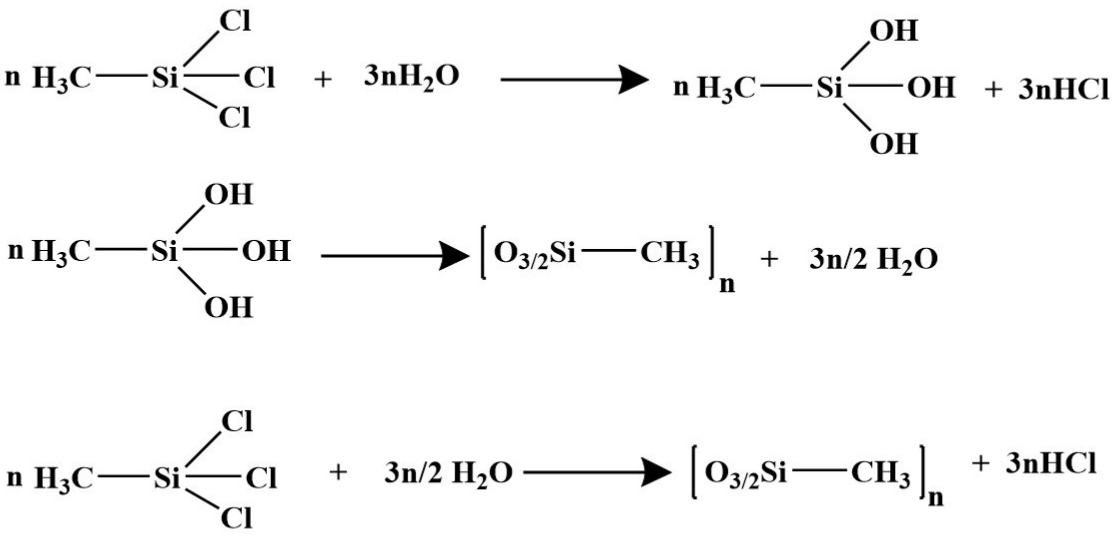

1D PSNFs

Figure S1 1D PSNFs Synthesis Mechanism [5]

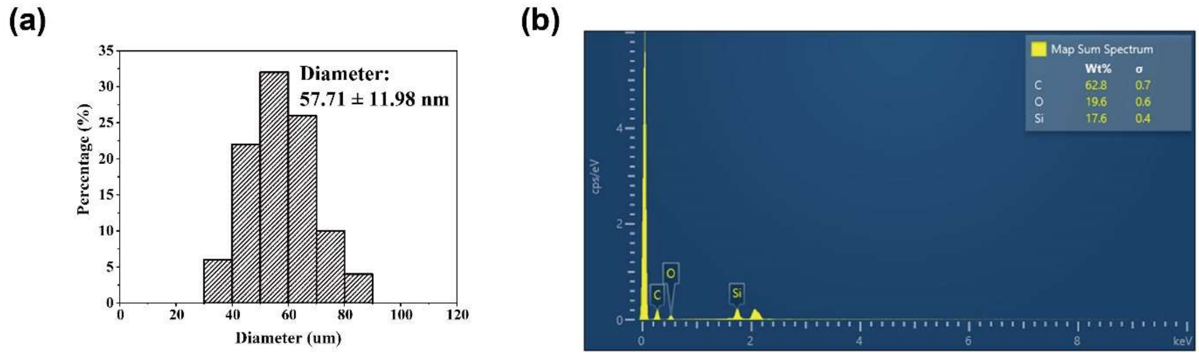

Figure S2 a. Diameter distribution of 1D PSNFs b. EDX elements mass distribution of the PSNF/MNPLA aerogel cooler with 20 wt.% 1D PSNFs

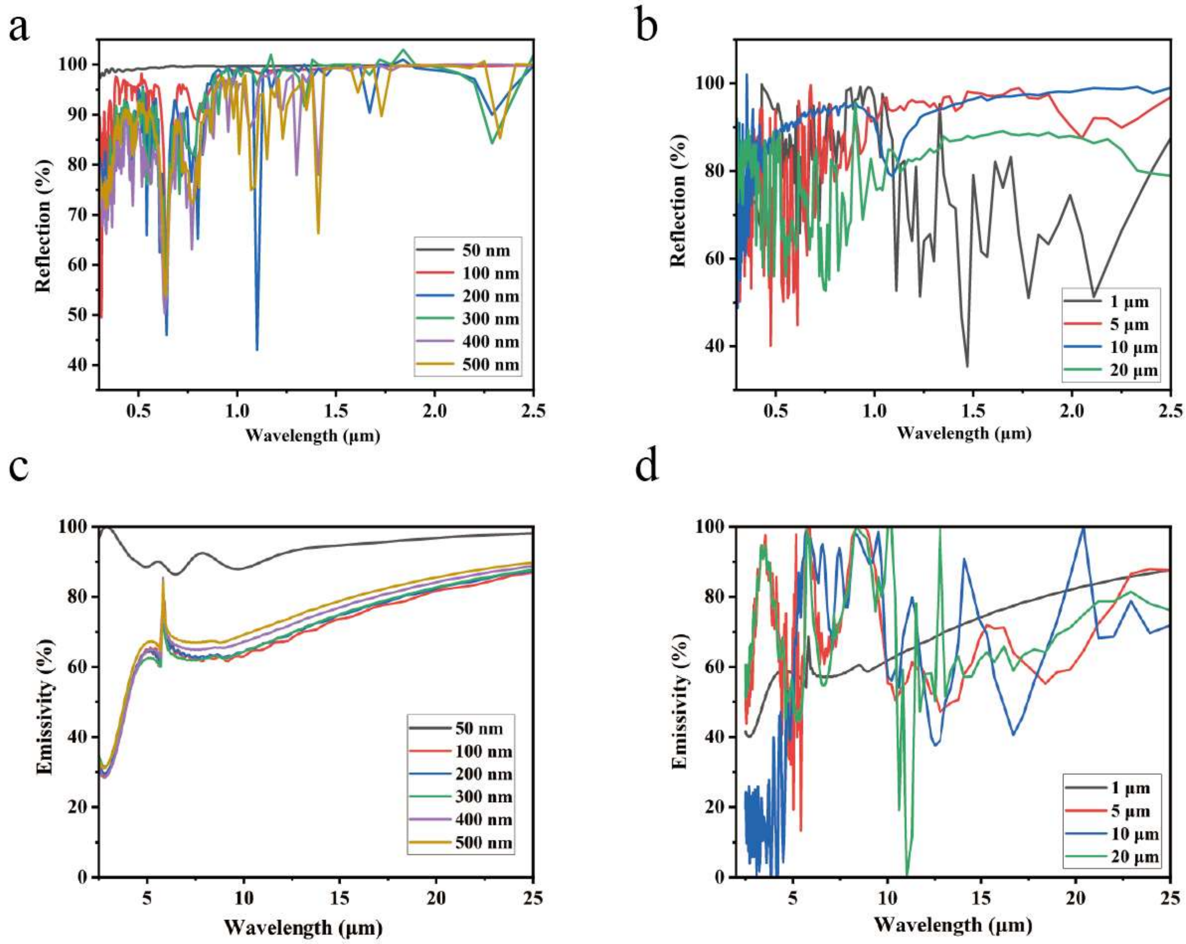

Figure S3. FDTD-simulated reflectance spectra of porous aerogels with different pore diameters in the UV–Vis–NIR (0.3–2.5 μm) and Mid-IR (2.5–25 μm) ranges. a) UV–Vis–NIR reflectance for nanopores ranging from 50 to 500 nm. b) UV–Vis–NIR reflectance for micropores ranging from 1 to 20 μm. c) Mid-IR reflectance for nanopores (50–500 nm). d) Mid-IR reflectance for micropores (1–20 μm).

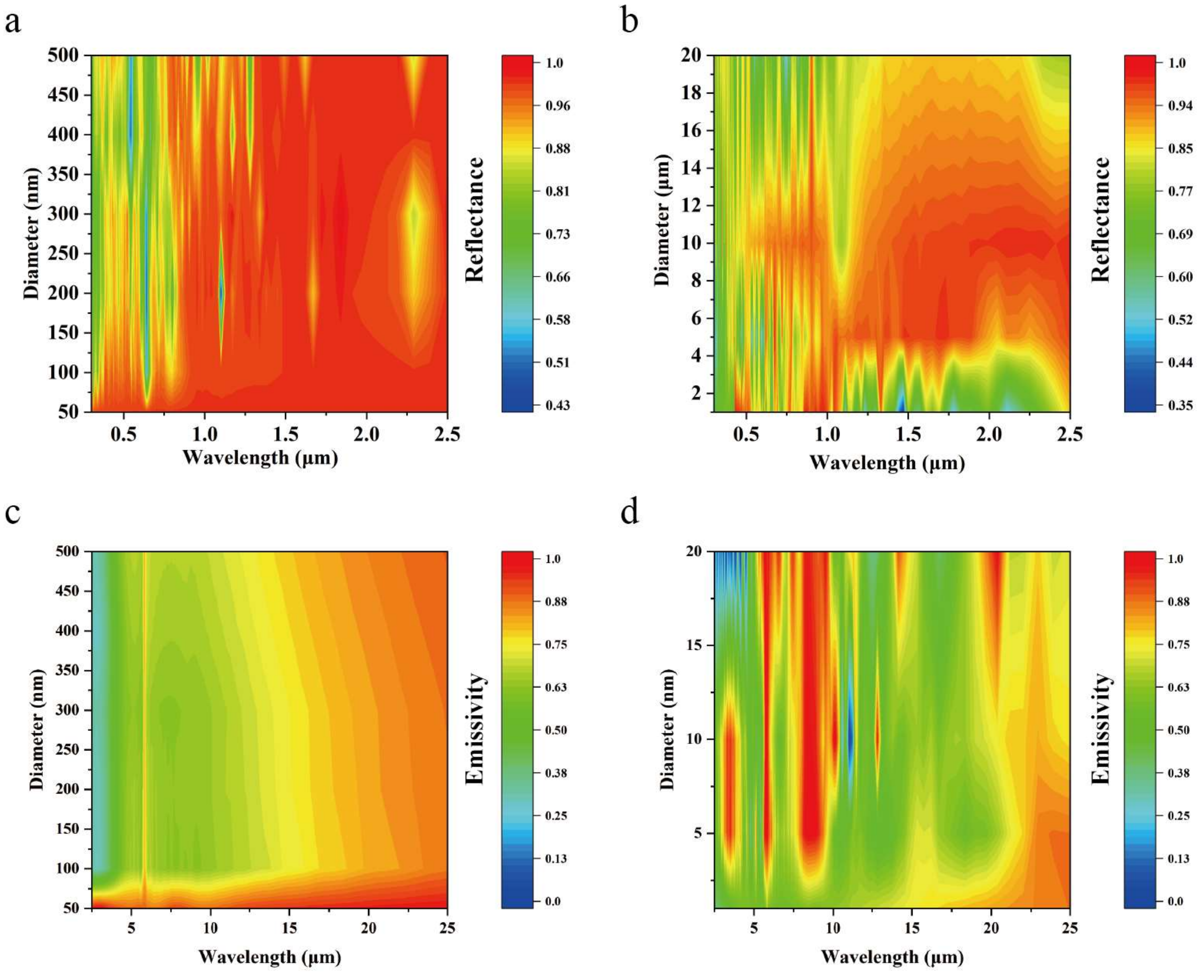

Figure S4. 2D heatmap representations of simulated reflectance spectra for aerogels with varying pore sizes using FDTD simulations. a) and b) show the spectral reflectance (0.3–2.5 μm) of nanoporous (50–500 nm) and microporous (1–20 μm) aerogels, respectively. c) and d) display the corresponding reflectance maps in the mid infrared range (2.5–25 μm).

To systematically evaluate the impact of pore size on spectral reflectance, we employed FDTD simulations and visualized the results as 2D heatmaps (**Figure S4**). These 2D heatmaps provide an intuitive visualization of how pore diameter influences wavelength-dependent optical responses across a broad spectral range. The intensity scale reveals distinct reflection regimes governed by resonant scattering and interference effects, highlighting the role of hierarchical porosity in tailoring solar reflection and infrared emissivity.

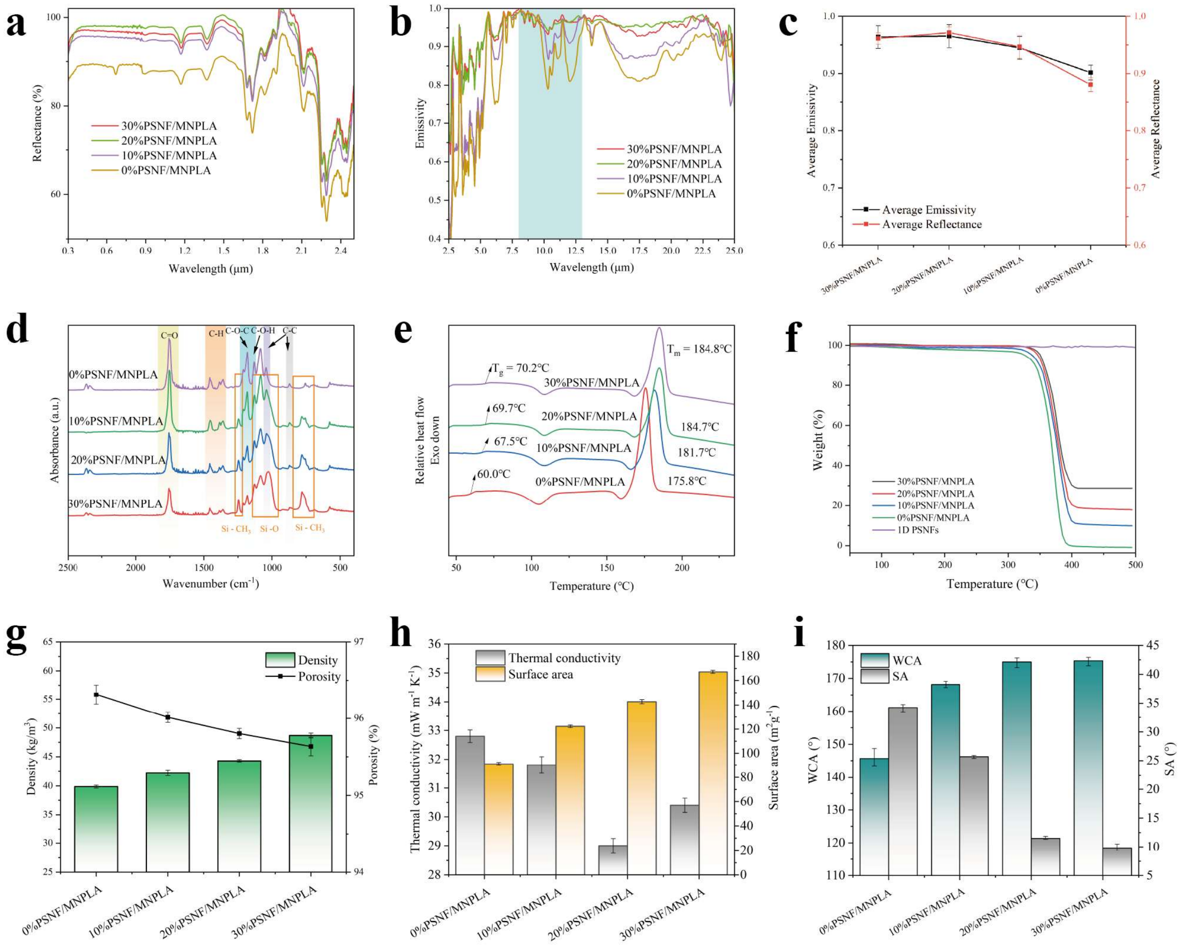

Figure S5 a) Reflectance, b) Emissivity, c) average reflectance and emissivity (8 – 13 μm), d) absorbance spectra, e) DSC curves, f) TGA curves, g) Density and porosity, h) Thermal conductivity and surface area, i) water contact angle of the aerogels with interspersing 0, 10%, 20%, and 30% 1D PSNFs

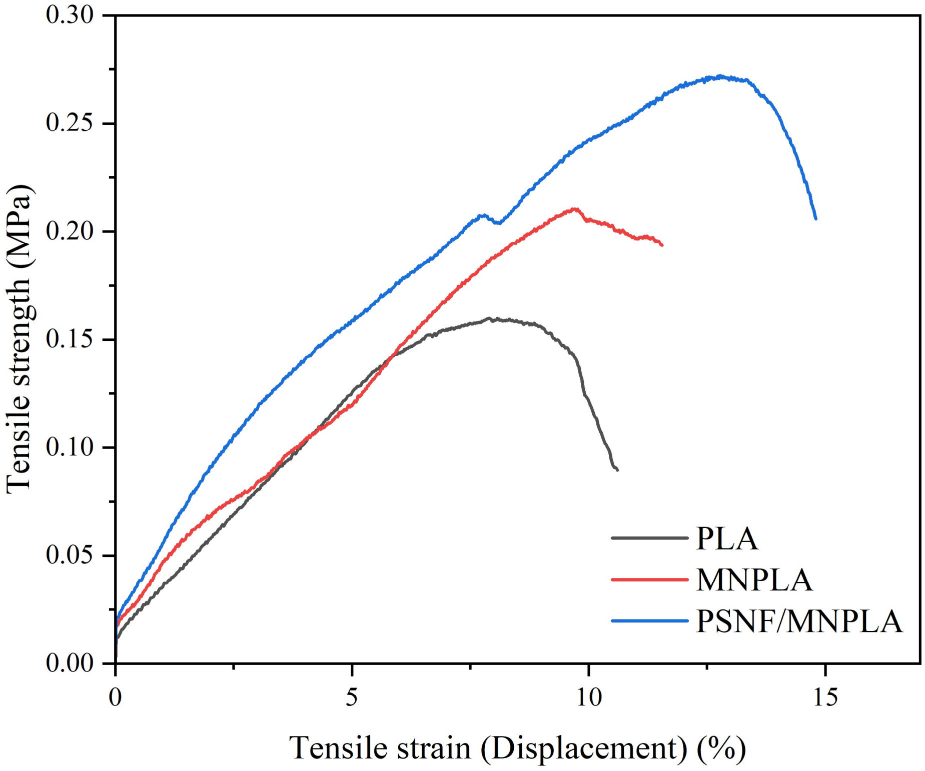

Figure S6 Tensile stress–strain curves for PLA, MNPLA, and PSNF/MNPLA

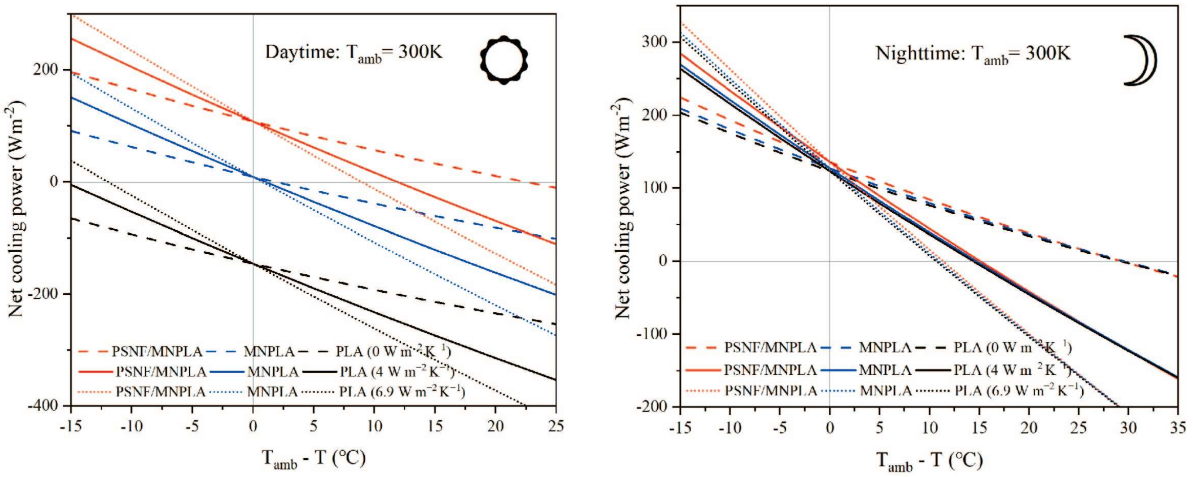

Figure S7 The cooling power of PLA, MNPLA, and PSNF/MNPLA aerogel coolers was computed for daytime (26.85°C, 300 K) and nighttime (26.85°C, 300 K) with h_c values of 0, 4 W m^{-2} K^{-1}, and 6.9 W m^{-2} K^{-1}.

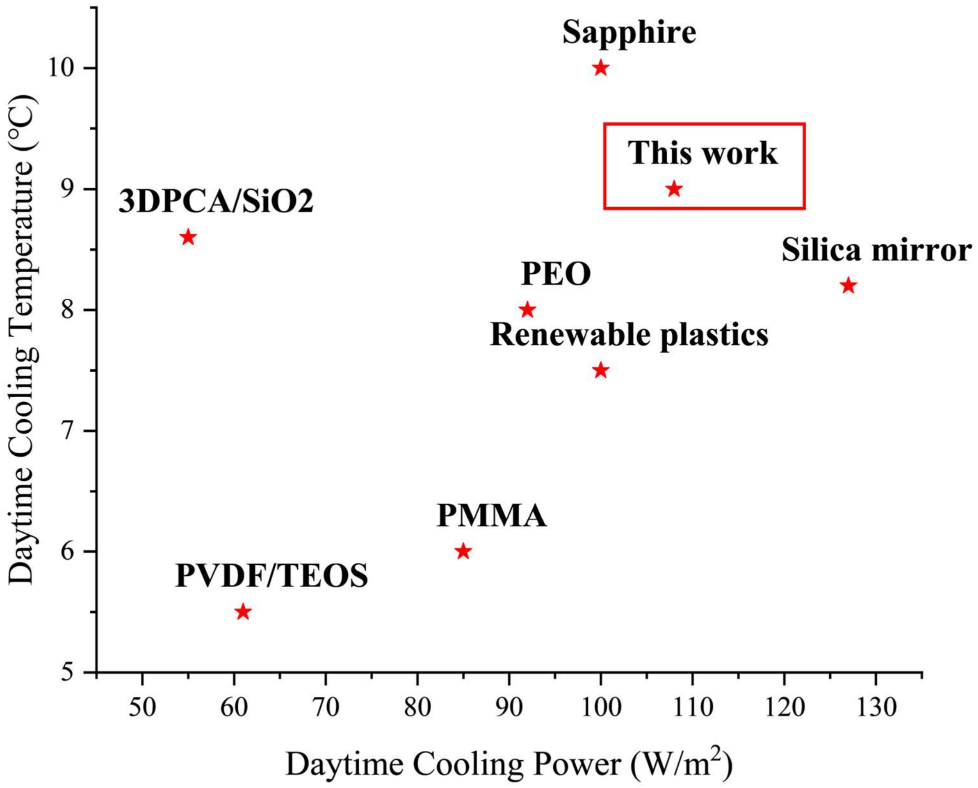

Figure S8 The comparation of PSNF/MNPLA with other coolers at the ambient temperature about 300 K

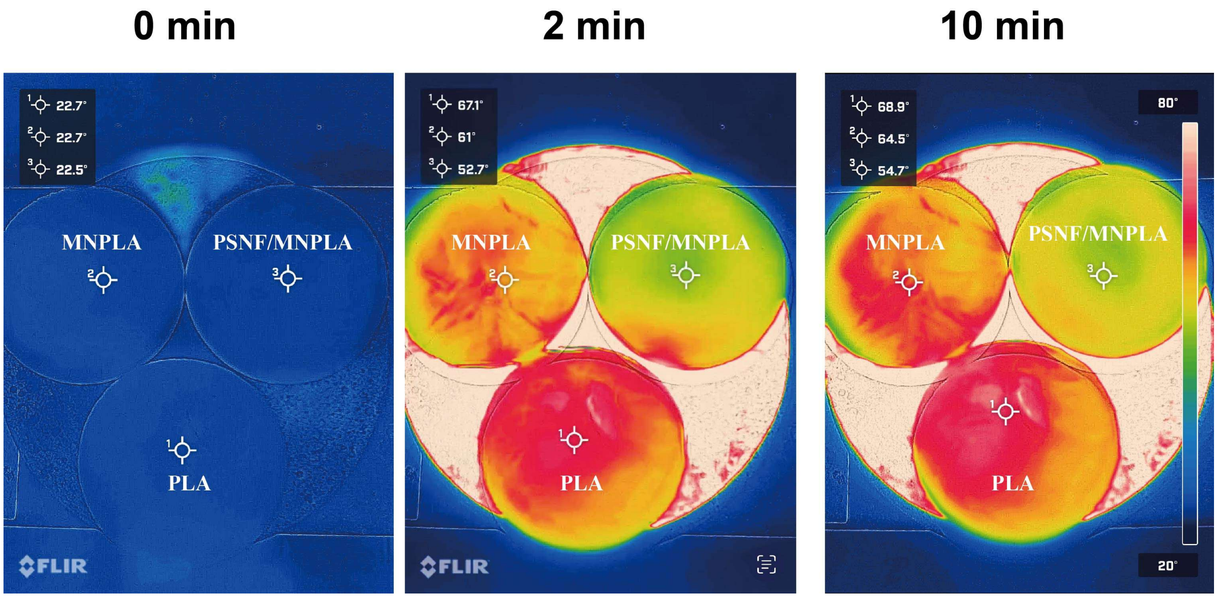

Figure S9 Infrared images of coolers on a hot plate set at 100°C for 10 minutes. Images at 0, 2 min, and 10 min.

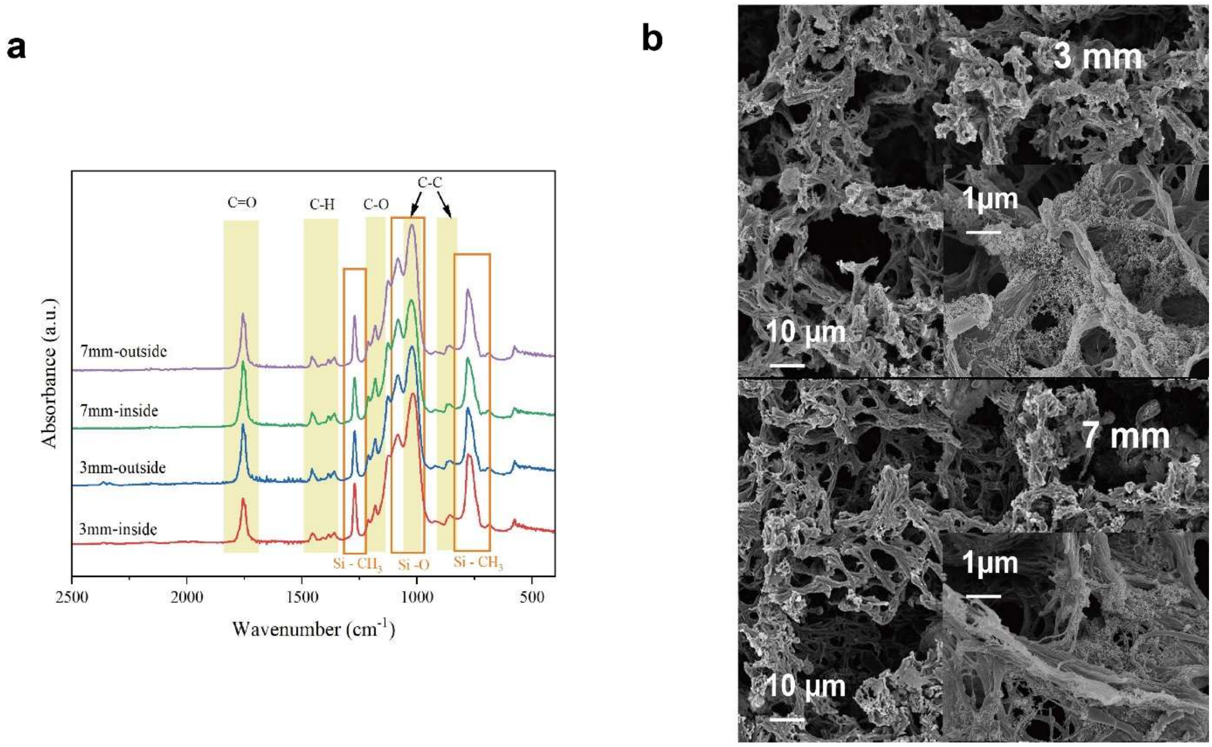

Figure S10 a) Absorbance and b) the SEM images of PSNF/MNPLA at different thicknesses (3mm and 7mm)

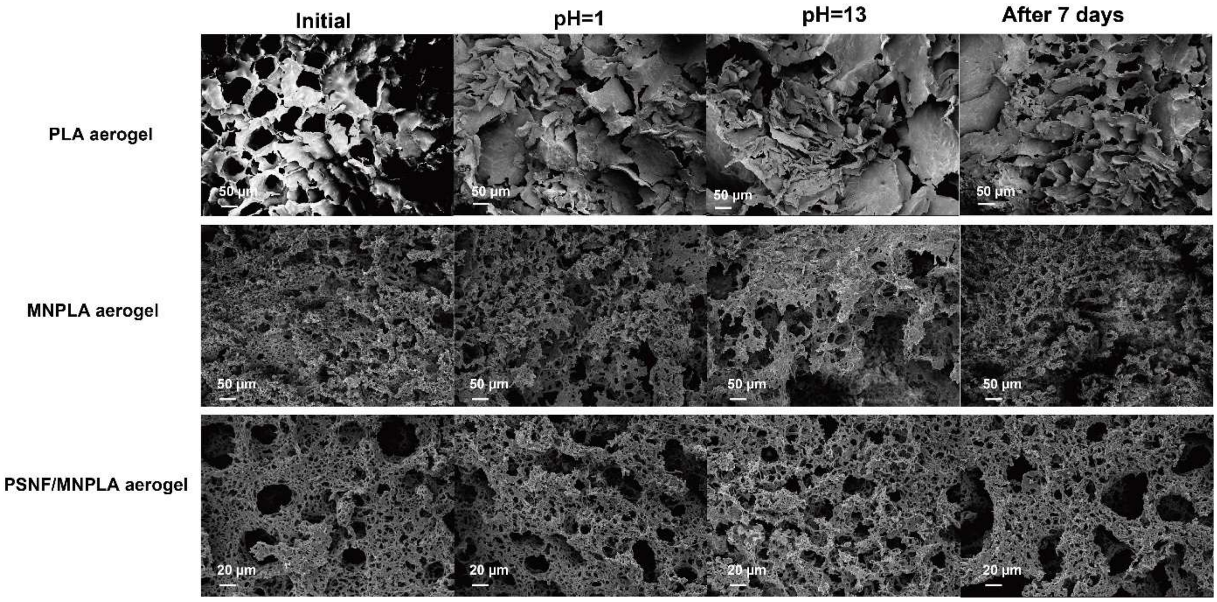

Figure S11 Morphology of PLA, MNPLA, and PSNF/MNPLA after 7 days corrosion in a solution of pH =1 and 13 and exposure under the summer sky.

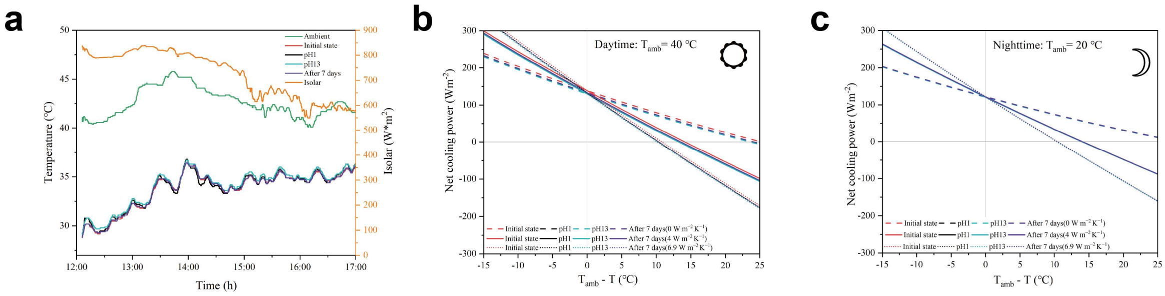

Figure S12 a) Temperature in the daytime (18 July 2023) in Zurich, Switzerland. b) and c) Calculated cooling power of PSNF/MNPLA aerogel cooler after 7-day corrosion in the solution of pH =1 and 13, and exposed under summer sky procedure as a function of cooling temperature for daytime nighttime with h_c = 0, 4 W m^{-2} K^{-1}and 6.9 W m^{-2} K^{-1}

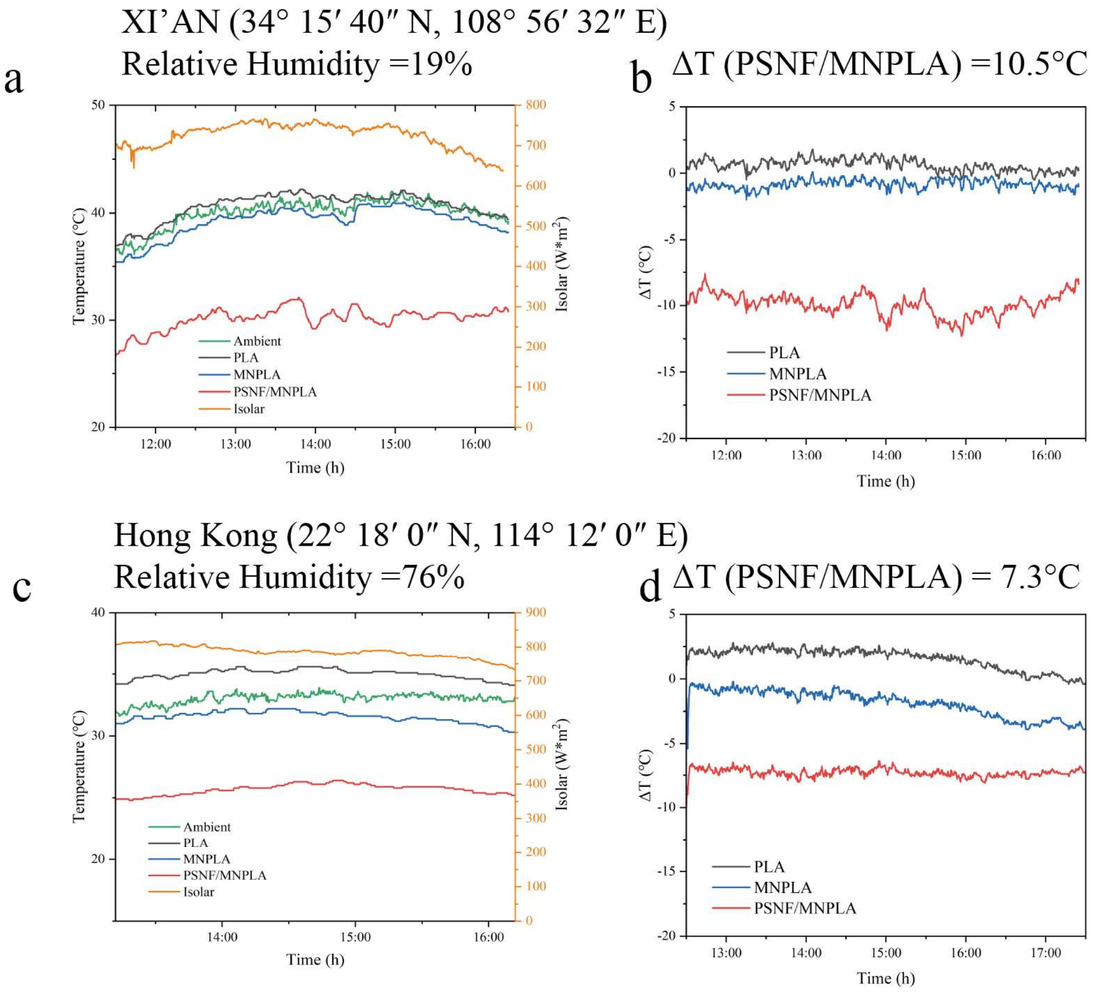

Figure. S13 Passive daytime radiative cooling performance of the aerogel coolers in different locations and weathers. a), Temperature, and b) temperature difference of PLA, MNPLA, and PSNF/MNPLA aerogels (conducted on May 1, 2025, in XI’AN, China, under clear skies and low wind conditions. During the test period, averaged ambient temperatures 39.8°C, with average relative humidity 19%.) c) Temperature, and d) temperature difference of PLA, MNPLA, and PSNF/MNPLA aerogels (conducted on May 4, 2025, in Hong Kong, China, under clear skies and low wind conditions. During the test period, averaged ambient temperatures 32.3°C, with average relative humidity 76%.)

To further investigate the robustness of the passive radiative cooling performance under different environmental conditions, we conducted additional outdoor tests in two geographically distinct locations: XI'AN (characterized by low humidity, 19%) and Hong Kong (high humidity, 76%), as shown in **Figure S13**. Despite the notable differences in ambient humidity and temperature, the PSNF/MNPLA aerogel consistently achieved significant sub-ambient cooling relative to PLA and MNPLA samples. Although the cooling performance slightly declined under the more humid conditions in Hong Kong, it remained substantial, demonstrating the aerogel cooler’s strong resilience and effectiveness across varied climatic environments.

Table S1. The thermal parameter of aerogel samples with various amounts of adding 1D PSNFs

	T_g (°C)	T_m (°C)	T_{d5} (°C)	T_{d10} (°C)	T_{max} (°C)	R_{500} (%)
PLA	53.3	171.8	212.1	335.3	374.4	0.02
MNPLA	60.0	175.8	325.7	341.1	371.6	0.09
10%PSNF/MNPLA	67.5	181.7	335.9	350.0	379.1	9.96
20%PSNF/MNPLA	69.7	184.7	345.9	353.2	379.4	17.91
30%PSNF/MNPLA	70.2	184.8	350.1	355.0	379.3	28.75

Table S2. The comparation of PSNF/MNPLA aerogel with other aerogel coolers

Aerogel coolers	Thickness/ mm	Cooling temperature/ °C	Reflectivity /%	Emissivity /%	Daytime cooling power W/m²	Thermal conductivity mW m^{-1} K^{-1}	Ref.
Polyethylene	6	13	92.2%	79.9%	96	28	6
GG-MF-SiO2	15	4.8	93%	94%	79.41	37	7
Cellulose nanocrystal	10	10.5	97.4 %	94 %	~75	29	8
Silica	5	18.6	98.1	92.1	103.3	~31	9
Photoluminescence-induced biomass	5	16.0	104.0%	90%	/	38.4	10
Polydimethylsiloxane	3	4.6	93	96	43	60	11
Cooling wood	200	>4	96	90	16	> 100	12
PVA/DIA@MPA-30	3.25	15.3	91.47	92.98	99.9	50	13
PSNF/MNPLA	3	9	97	97	138.6	29	This work

References

(1) Wang, T.; Wu, Y.; Shi, L.; Hu, X.; Chen, M.; Wu, L. A structural polymer for highly efficient all-day passive radiative cooling. *Nat Commun* **2021**, *12* (1), 365.
(2) Gao, W.; Lei, Z.; Wu, K.; Chen, Y. Reconfigurable and Renewable Nano-Micro-Structured Plastics for Radiative Cooling. *Advanced Functional Materials* **2021**, *31* (21).
(3) Raman, A. P.; Anoma, M. A.; Zhu, L.; Rephaeli, E.; Fan, S. Passive radiative cooling below ambient air temperature under direct sunlight. *Nature* **2014**, *515* (7528), 540-544.
(4) Hossain, M. M.; Gu, M. Radiative Cooling: Principles, Progress, and Potentials. *Adv Sci (Weinh)* **2016**, *3* (7), 1500360. Xiang, B.; Zhang, R.; Luo, Y.; Zhang, S.; Xu, L.; Min, H.; Tang, S.; Meng, X. 3D porous polymer film with designed pore architecture and auto-deposited SiO2 for highly efficient passive radiative cooling. *Nano Energy* **2021**, *81*. DOI: 10.1016/j.nanoen.2020.105600.
(5) Artus, G. R.; Seeger, S. One-dimensional silicone nanofilaments. *Adv Colloid Interface Sci* **2014**, *209*, 144-162. DOI: 10.1016/j.cis.2014.03.007.
(6) Leroy, A.; Bhatia, B.; Kelsall, C. C.; Castillejo-Cuberos, A.; Di Capua H., M.; Zhao, L.; Zhang, L.; Guzman, A. M.; Wang, E. N. High-performance subambient radiative cooling enabled by optically selective and thermally insulating polyethylene aerogel. *Science Advances* **2019**, *5* (10), eaat9480. DOI: doi:10.1126/sciadv.aat9480.
(7) Peng, Z. C.; Zeng, F. R.; Zeng, Z. W.; Su, P. G.; Tang, P. J.; Liu, B. W.; Zhang, Y.; Wang, Y. Z.; Zhao, H. B. Scalable Low-Carbon Ambient-Dried Foam-Like Aerogels for Radiative Cooling with Extreme Environmental Resistance. *Adv Mater* **2025**, e2505224. DOI: 10.1002/adma.202505224.
(8) Cai, C.; Chen, W.; Wei, Z.; Ding, C.; Sun, B.; Gerhard, C.; Fu, Y.; Zhang, K. Bioinspired “aerogel grating” with metasurfaces for durable daytime radiative cooling for year-round energy savings. *Nano Energy* **2023**, *114*. DOI: 10.1016/j.nanoen.2023.108625.
(9) Lan, P. H.; Hwang, C. W.; Chen, T. C.; Wang, T. W.; Chen, H. L.; Wan, D. Hierarchical Ceramic Nanofibrous Aerogels for Universal Passive Radiative Cooling. *Advanced Functional Materials* **2024**, *34* (52). DOI: 10.1002/adfm.202410285.
(10) Ma, J.-W.; Zeng, F.-R.; Lin, X.-C.; Wang, Y.-Q.; Ma, Y.-H.; Jia, X.-X.; Zhang, J.-C.; Liu, B.-W.; Wang, Y.-Z.; Zhao, H.-B. A photoluminescent hydrogen-bonded biomass aerogel for sustainable radiative cooling. *Science* **2024**, *385* (6704), 68-74. DOI: doi:10.1126/science.adn5694.
(11) Zhou, L.; Rada, J.; Zhang, H.; Song, H.; Mirniaharikandi, S.; Ooi, B. S.; Gan, Q. Sustainable and Inexpensive Polydimethylsiloxane Sponges for Daytime Radiative Cooling. *Adv Sci (Weinh)* **2021**, *8* (23), e2102502.
(12) Li, T.; Zhai, Y.; He, S.; Gan, W.; Wei, Z.; Heidarinejad, M.; Dalgo, D.; Mi, R.; Zhao, X.; Song, J.; et al. A radiative cooling structural material. *Science* **2019**, *364* (6442), 760-763.
(13) Yin, L.; Zhang, J.; Luo, J.; Shi, C.; Qian, X.; Yu, B.; Zhang, S.; Zhou, K. Fire safety protective polyvinyl alcohol-based “green” aerogels with micro/nano dual-scale porous structure for passive radiative cooling. *Chemical Engineering Journal* **2025**, *512*. DOI: 10.1016/j.cej.2025.162530.
